# Activation of the antibiotic resistance factor WhiB7 can stimulate aggregate biofilm formation in stationary phase *Mycobacterium smegmatis* by reinitiating translation

**DOI:** 10.1128/jb.00076-26

**Published:** 2026-06-22

**Authors:** Mitchell D. Meyer, Megan Bergkessel, William H. DePas

**Affiliations:** 1 Department of Pediatrics, University of Pittsburgh School of Medicine 12317, Pittsburgh, USA; 2 Division of Molecular Microbiology, School of Life Sciences, University of Dundee 98264, Dundee, United Kingdom; University of Notre Dame, Notre Dame, Indiana, USA

**Keywords:** two-component systems, dormancy, antibiotic tolerance, NTM, biofilms

## Abstract

There is a growing understanding that slow growth and dormancy due to nutrient deprivation are very common physiological states exhibited by bacterial communities in a myriad of environments. However, very little is known about the role of slow growth and dormancy in biofilm regulation. Here, we utilize tractable dormancy and aggregation assays in nontuberculous mycobacteria (NTM) to ask the fundamental question of how growth arrest impacts the processes of aggregation and dispersal. First, we show that the well-conserved dormancy regulator DosSR affects biofilm formation in *Mycobacterium smegmatis*, as a *dosR* deletion mutant undergoes spurious re-aggregation and dispersal during aerobic late stationary phase. Identification of a suppressor mutation blocking re-aggregation in the Δ*dosR* mutant allowed us to determine a role for the antibiotic resistance factor WhiB7 in driving re-aggregation in *M. smegmatis*. We utilized BioOrthogonal NonCanonical Amino acid Tagging (BONCAT), qPCR, and quantitative aggregation assays to build a model wherein reductive stress in Δ*dosR* potentiates stationary phase translation in a WhiB7-dependent manner, permitting aggregation in dormant stationary phase cells. In addition, during stationary phase, WhiB7-activating reducing agents and antibiotics could trigger re-aggregation in both wild-type *M. smegmatis* and clinical isolates of the opportunistic NTM pathogen *Mycobacterium abscessus*. Finally, we determined that, in contrast to aerobic stationary phase, *M. smegmatis* does not aggregate or disperse in response to chemical cues or antibiotics under the Wayne model of hypoxic dormancy. Our work reveals a regulatory interaction between dormancy and aggregation that could have broad implications for treating and preventing NTM biofilms.

**IMPORTANCE:**

Mycobacteria aggregate to form multicellular biofilms that provide protection from external stressors and increase antibiotic tolerance. Understanding the pathways regulating biofilm formation can aid the identification of useful targets for developing new drugs. With a growing appreciation that pathogens are often in a slow growth/dormant state during infection, we investigate how dormancy affects biofilm formation and dispersal in two nontuberculous mycobacteria (NTM) species: *Mycobacterium smegmatis* and the opportunistic pathogen *Mycobacterium abscessus*. We find that activation of the WhiB7-mediated antibiotic response permits biofilm formation in aerobic stationary phase by reinitiating protein synthesis; however, cells under hypoxic dormancy are unresponsive. Our work provides important context to combatting biofilm formation in infection sites, informing future studies and aiding design of biofilm dispersal agents.

## INTRODUCTION

In the last 50 years, there has been a growing appreciation that the majority of bacteria on Earth likely exist in some type of biofilm (1). This paradigm has supported and motivated numerous fruitful studies investigating the regulatory systems governing multicellular bacterial community development. More recently, *in situ* measurements from host and nonhost environments (2–6) have revealed that dormancy/slow growth is a prevalent *in situ* physiological state, and the liquid culture exponential growth we typically utilize in the laboratory is likely an exception rather than the rule (3, 5). Accordingly, determining how biofilm formation and dormancy interact, specifically how dormancy affects biofilm formation and dispersal, is a fundamental question with broad implications to most bacteria on the planet. Previous studies, including our own, have focused on understanding the regulatory mechanisms governing aggregation and dispersal in the context of metabolically active biofilms (7–9). When dormancy is considered in relation to biofilms, it is almost always considered to be derived from the biofilm structure itself, i.e., interior regions becoming dormant due to the formation of nutrient gradients (10). However, very little is known about biofilm regulation within bacterial populations composed entirely of dormant cells and the regulatory consequences of entire biofilm communities becoming starved and driven into dormancy.

NTM colonize diverse niches including soil and waterways, the built environment, and human and animal hosts (11–15). The ability of NTM to persist within these disparate environments is dependent, in part, on biofilm formation (11, 12). Indeed, microscopy of *ex vivo* lung tissue and CF sputum from infected patients shows *Mycobacterium abscessus* (MABS) growing as biofilm aggregates during infection (16, 17). Here, we investigate how dormancy affects the ability of NTM to enter and disperse from aggregated biofilm growth. NTM undergo a well-studied programmed dormancy response, largely coordinated by the DosSR two-component system, which is conserved across mycobacteria (18–21). DosS is a sensor histidine kinase activated by hypoxia, NO, or a disruption to redox balance that then phosphorylates the transcriptional regulator DosR (22–25). DosSR is essential for survival in hypoxic conditions and participates in maintaining metabolic and redox homeostasis (18, 26–28). While primarily considered in the context of hypoxia, *dosR* is also upregulated in aerobic conditions which lead to growth arrest, like aerobic stationary phase (20, 21). Many DosR regulon components affect metabolic adaptation to hypoxic and/or slow growth environments, including synthesis of storage lipids, hydrogen metabolism, universal stress proteins, and ribosome-associating hibernation promotion factors (HPFs) like RafH (18, 21, 24, 29, 30).

To better understand the connection between biofilm formation, dispersal, and dormancy, we utilized our tractable *in vitro* aggregation assay in the model NTM *Mycobacterium smegmatis* in conjunction with two distinct models of dormancy (9). NTM liquid culture aggregation is dependent on cell-cell adhesion, a critical early stage in NTM biofilm development, and it can be used as a proxy to determine factors which contribute to biofilm formation and dispersal (7, 9). While various causes of growth arrest can converge on and activate master regulators like DosSR, a broad demarcation can be drawn between nutrient limitation and oxidant starvation. Cellular physiology and survival requirements differ between the two states, and in some bacteria like *Pseudomonas aeruginosa*, oxygen starvation more severely decreases ATP pools compared to carbon starvation (27, 31). We, therefore, assessed aggregation in aerobic stationary phase (nutrient limitation) and Wayne model hypoxia (oxygen limitation), where cells are grown in rubber-septum sealed Balch tubes and cultures slowly run out of oxygen as they consume the headspace O_2_ (32).

Our results support a model in which the DosSR system helps to suppress aggregation in aerobic stationary phase. However, in the absence of *dosR*, reductive stress leads to the activation of WhiB7, which permits aggregation and dispersal to proceed by reinitiating translation. Interestingly, direct chemical activation of WhiB7 with the clinically relevant antibiotic clarithromycin can also induce aggregation in *M. smegmatis* and *M. abscessus* in aerobic stationary phase. As dormancy and biofilm formation inevitably intersect in various bacterial species across numerous environments, we hope that understanding how these systems are interconnected in NTM will lay the groundwork for future studies in a variety of systems.

## RESULTS

### A *dosR* knock-out mutant shows dysregulated aggregation in aerobic stationary phase

**Fig 1:**
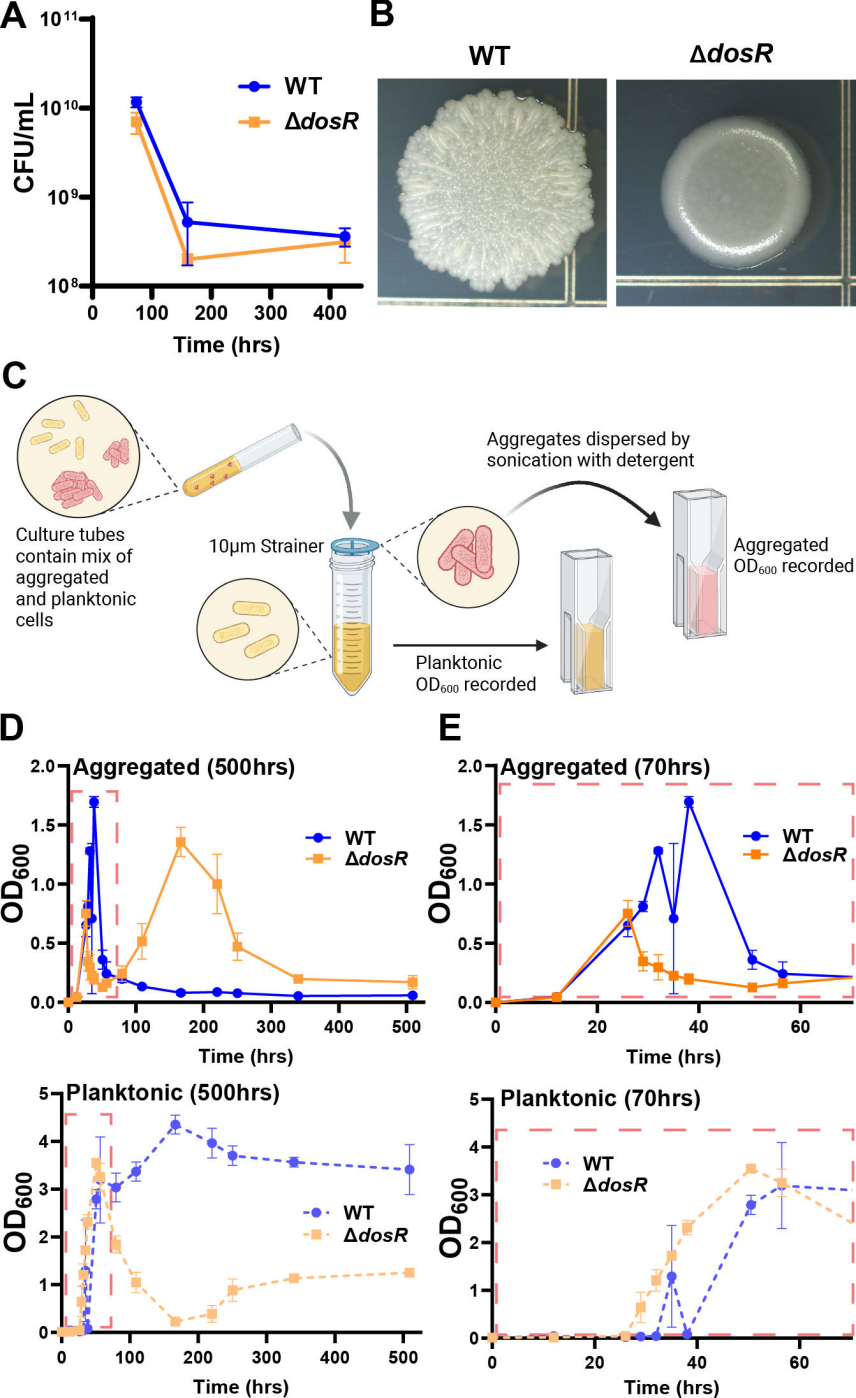
Deletion of the dormancy regulator *dosR* dysregulates aggregation in late stationary phase. (**A**) CFUs/mL of WT and Δ*dosR* during late aerobic stationary phase. (**B**) Colony morphology of rough WT *M. smegmatis* and the smooth Δ*dosR* mutant. (**C**) Graphical diagram of *in vitro* aggregation assay. Briefly, culture tubes of strain of interest were poured over a 10 µm strainer, which retained aggregated cells and allowed planktonic cells to pass through. Planktonic OD_600_ was quantified, and aggregated cells were washed with PBS before sonication with Tween80 and quantification of the dispersed aggregated cells. (**D**) Cultures of WT or Δ*dosR* were grown aerobically in rich medium (TYEM), and aggregate and planktonic fractions were quantified at indicated timepoints. (**E**) Initial aggregation window (dotted red rectangle in panel **D**). Each point represents the average of three technical replicate cultures, with the error bars indicating the standard deviation. Panel **C** was created in Biorender. Meyer, M. (2026) https://BioRender.com/c85iyta.

DosSR is one of the major regulators of dormancy in mycobacteria, and it is critical for survival under hypoxia (18, 26–28). To determine how regulation by the DosSR two-component system affects aggregation, we created a Δ*dosR* mutant in *M. smegmatis* mc^2^155 and evaluated its ability to form biofilms through use of our *in vitro* aggregation assay. We evaluated aggregation in late aerobic stationary phase, a condition in which increased expression of DosR regulon components is also observed but in which Δ*dosR* survives (20, 21, 27, 33). To determine the feasibility of profiling our Δ*dosR* mutant in late stationary phase, we first confirmed that our Δ*dosR* mutant was viable during aerobic stationary phase in rich tryptone yeast extract MgSO4 (TYEM) medium. CFU analysis through late stationary phase (425hr) demonstrated a similar survival between Δ*dosR* and WT *M. smegmatis* as reported previously (21), albeit with a small decrease in CFUs around 160hrs (Fig. 1A). In comparison, in the Wayne model of hypoxia, the Δ*dosR* mutant rapidly loses viability (Fig. S1). TYEM is compositionally similar to lysogeny broth (LB), and *E. coli* enters stationary phase in LB primarily due to carbon starvation (34). Alkaline stress due to ammonium excretion after amino acid catabolism can also limit growth in LB, and indeed, we have previously shown that *M. smegmatis* secretes excess ammonium in TYEM (7, 34, 35). To test whether the same trends held true for *M. smegmatis* in TYEM, we grew *M. smegmatis* in TYEM with nutrient supplements and monitored the OD_600_ at entrance into stationary phase (108 h). Pyruvate supplementation resulted in a slight but not-significant increase to the aggregate fraction OD_600_ (*P*-value = 0.13) while maintaining comparable planktonic fraction OD_600_, indicating that carbon depletion may be partially responsible for growth arrest in TYEM (Fig. S2A). In addition, TYEM buffered with MOPS (3-(N-Morpholino)propanesulfonic acid) to a pH of 7.2 supported a significantly higher final OD_600_ compared to baseline (Fig. S2A), consistent with alkaline stress inhibiting growth in stationary phase. MOPS also succeeded in lowering the final culture pH (Fig. S2B). No other nutrient supplementation increased stationary phase OD_600_ (Fig. S2A), indicating that alkaline stress and potentially carbon limitation are factors driving *M. smegmatis* growth arrest in our aerobic stationary phase model.

Next, we set out to probe aggregation dynamics of WT and Δ*dosR* in aerobic stationary phase in TYEM. We first noted that when plating Δ*dosR*, the mutant demonstrated a smooth colony morphology (Fig. 1B), typically indicative of altered biofilm formation (7, 36). Next, we assessed Δ*dosR* aggregation dynamics in aerobic stationary phase with a culture tube-based aggregation assay (Fig. 1C) and noted a striking feature. Unlike WT *M. smegmatis,* which stays planktonic after initial dispersal, the Δ*dosR* mutant underwent a dramatic re-aggregation and re-dispersal during late aerobic stationary phase (Fig. 1D). Indeed, the small drop in Δ*dosR* CFUs during re-aggregation at 160 h (Fig. 1A) could be due to the difficulty of fully breaking up mycobacterial aggregates, which can result in an artificial drop in CFUs (37). We also noted decreased aggregation of Δ*dosR* in the initial window (Fig. 1E). For the purposes of this study, we focused on the re-aggregation phenotype (from here forward re-aggregation and re-dispersal refer to events after the initial dispersal window of 60 h) due to the involvement of DosR in stationary phase. Complementation of *dosR* on the integrative pMH94 plasmid resulted in partial suppression of the re-aggregation phenotype (Fig. S3). These results suggest that DosR likely suppresses a pro-aggregation pathway in aerobic stationary phase in WT *M. smegmatis*, and that in the Δ*dosR* mutant, this signal proceeds unabated, leading to the re-aggregation phenotype.

### A suppressor mutant in the Δ*dosR* background contains a mutation in the uORF of *whiB7*

**Fig 2:**
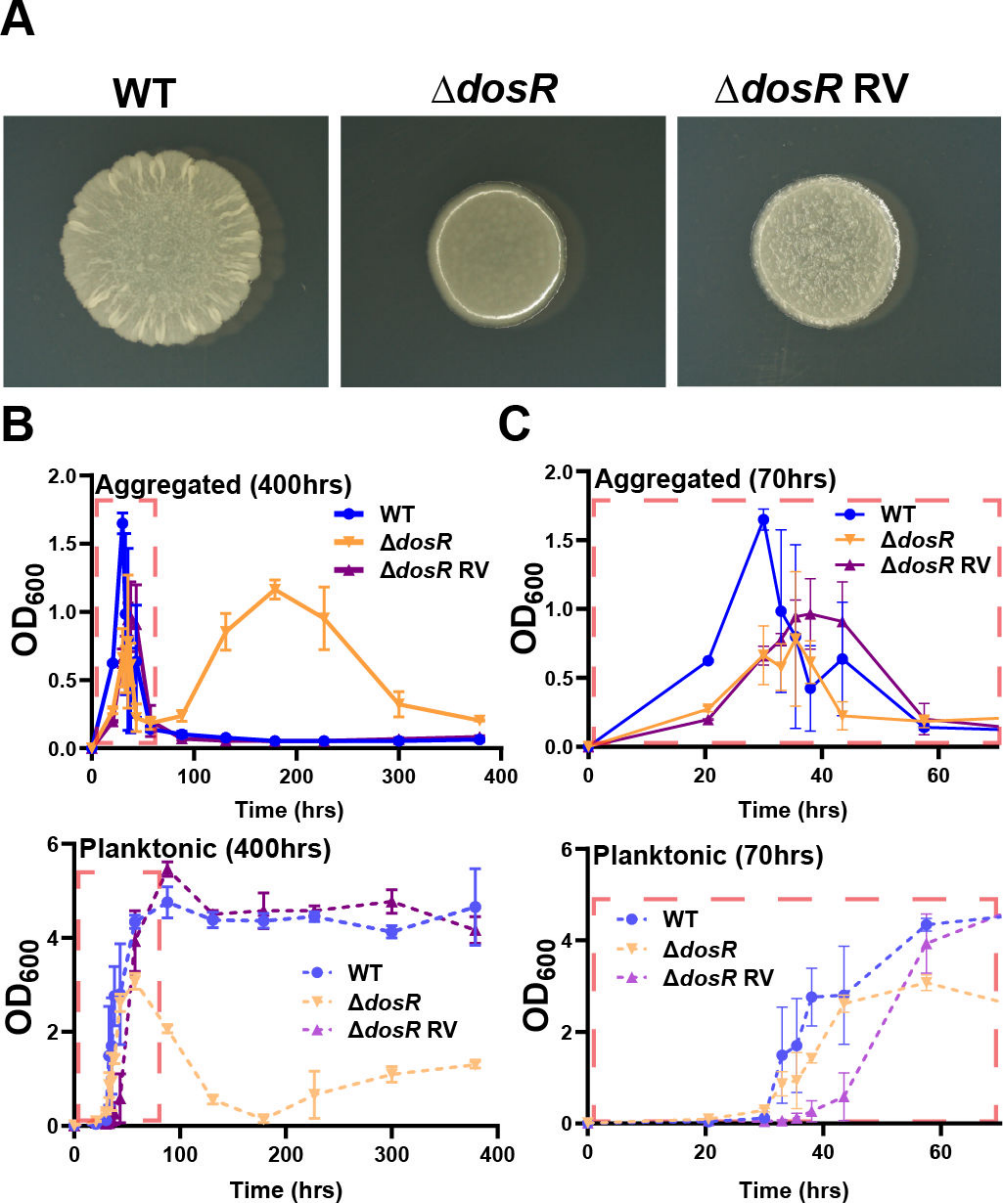
A Δ*dosR* rough colony variant no longer undergoes re-aggregation. (**A**) A Δ*dosR* suppressor mutant with rough colony morphology (Δ*dosR* RV) in comparison to WT *M. smegmatis* and the initial smooth colony Δ*dosR* mutant. (**B**) Aggregation assay in TYEM of WT *M. smegmatis* in comparison to Δ*dosR* and Δ*dosR* RV during late stationary phase (400 h) and (**C**) initial aggregation window (70 h), the dotted red rectangle in Fig. 2B. Each point represents the average of three technical replicate cultures, with the error bars indicating the standard deviation.

To obtain insight into the systems affecting re-aggregation in the Δ*dosR* mutant, we sought out a suppressor mutant that had reduced re-aggregation in aerobic stationary phase. We utilized the smooth colony morphology of the Δ*dosR* mutant to visually screen serial passaged colonies for a wrinkled colony morphology similar to WT *M. smegmatis*. With this methodology, we were able to identify and isolate a variant of Δ*dosR* (Δ*dosR* RV), with an intermediate morphology which was rougher than the original Δ*dosR* mutant (Fig. 2A). We assessed the aggregation dynamics of Δ*dosR* RV and found that it no longer displayed the characteristic re-aggregation and re-dispersal of the Δ*dosR* mutant in aerobic stationary phase (Fig. 2B and C). With the expectation that Δ*dosR* RV had obtained a suppressor mutation, we sequenced the genomes of the Δ*dosR* and Δ*dosR* RV mutants. The only mutation unique to Δ*dosR* RV was a G->A mutation 390 bp upstream of, and within the upstream open reading frame (uORF) region of, *whiB7*, a well-studied transcription factor known to be involved in mycobacterial antibiotic resistance and redox homeostasis (38–40). We therefore set out to explore what effect WhiB7 might be imposing on aggregation in the context of slow growth and dormancy.

### *whiB7* induction is necessary for re-aggregation in the Δ*dosR* background

**Fig 3:**
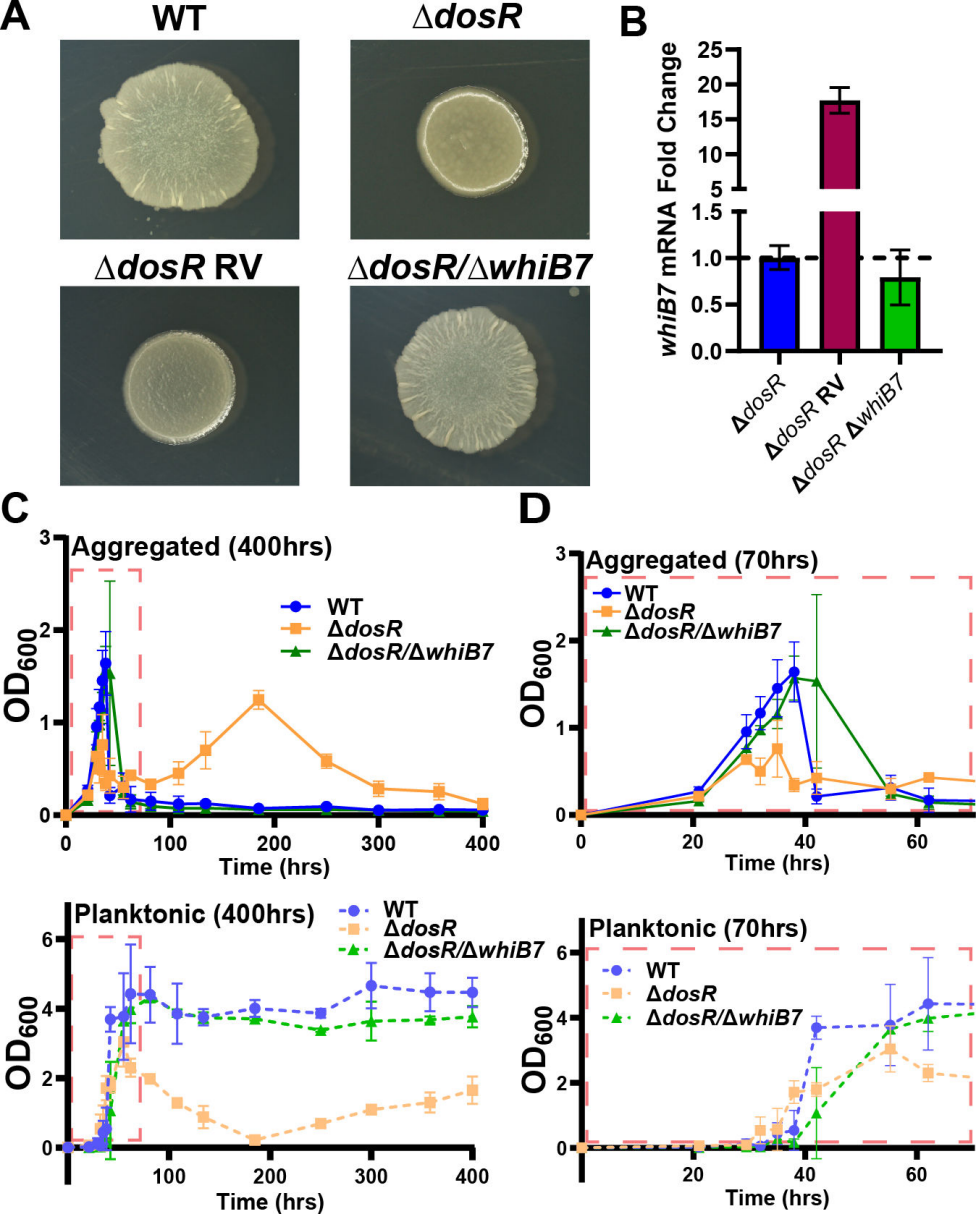
Presence of *whiB7* is necessary to allow re-aggregation, but *whiB7* overexpression can also block re-aggregation in the Δ*dosR* mutant. (**A**) A Δ*dosR*/Δ*whiB7* mutant fully restored WT rough colony morphology. (**B**) Fold change in *whiB7* mRNA above WT by qPCR, from triplicate WT, Δ*dosR*, Δ*dosR* RV, Δ*dosR*/Δ*whiB7* cultures during stationary phase (160 h). Expression levels were normalized against the housekeeping gene *mysA*, and WT mRNA was used as the non-experimental control. (**C**) Aggregation assay in TYEM of WT vs Δ*dosR* vs Δ*dosR*/Δ*whiB7* in the re-aggregation window (400 h) and (**D**) the initial aggregation window (70 h), represented by the dotted red rectangle in Fig. 3C. Each point represents the average of three technical replicate cultures, with the error bars indicating the standard deviation.

Mutations to the upstream open reading frame uORF can affect the expression of *whiB7* (40, 41). To first test the role of WhiB7 in Δ*dosR* re-aggregation*,* we constructed a clean Δ*whiB7* knockout mutant in the Δ*dosR* background through recombineering. The resulting Δ*dosR*/Δ*whiB7* double knockout mutant displayed a similar colony morphology to WT (Fig. 3A), and no longer re-aggregated during aerobic stationary phase (Fig. 3C), similar to Δ*dosR* RV. We assessed *whiB7* transcript levels in WT, Δ*dosR,* Δ*dosR* RV, and Δ*dosR*/Δ*whiB7* using qPCR. WT, Δ*dosR,* and Δ*dosR*/Δ*whiB7* all had low levels of *whiB7* transcript signal in late aerobic stationary phase (~34 adjusted Ct), with the double mutant Ct values at the threshold of detection (34.8 Ct) (Fig. 3B; Table S2). Surprisingly, the Δ*dosR* RV mutant had substantially increased *whiB7* expression compared to WT (29.4 Ct) (Fig. 3B). At first, these two results seemed contradictory, but literature review revealed a model wherein it was plausible that too much *whiB7* expression (Δ*dosR* RV) or no *whiB7* expression (Δ*dosR*/Δ*whiB7*) could both suppress re-aggregation. The WhiB7-mediated antibiotic response is a well-studied system within Actinobacteria, where it can increase antibiotic tolerance by inducing systems including *tap* drug efflux pumps, the *eis* drug-targeting acyltransferase, and, critically, the ribosome modifying methyltransferase *erm* and ribosome splitting proteins such as HflX (39, 42–44). WhiB7-mediated ribosome splitting rescues ribosomes stalled by antibiotics or nutritional stress, causing dissociation and disordering of subunits, and can allow translation to resume when conditions improve at a later time, such as after abatement of intracellular antibiotic or with reintroduction of nutrients (38, 39, 42–46). If WhiB7-mediated resumption of translation is necessary for aggregation and dispersal in stationary phase, that could explain why our Δ*dosR*/Δ*whiB7* mutant fails to re-aggregate.

We posit that too much of this response may have the opposite effect, as the ribosome splitting and 23S rRNA disordering effected by *M. smegmatis* HflX causes the 50S LSUs to remain inactive even after HflX release, requiring ribosome biogenesis factors in order to return to their active form (42, 43, 45). WhiB7 also regulates the *M. smegmatis erm* homolog *erm*(38) as a part of its 96 gene regulon (46, 47). The encoded product Erm dimethyltransferase methylates the 23S rRNA, blocking macrolide or other ribosome-targeting antibiotics from binding, but in the absence of antibiotic, this activity has been shown to decrease translation efficiency and increase fitness cost in other species (46, 48–51). The overexpression of the WhiB7 regulon in the Δ*dosR* RV mutant during aerobic stationary phase could, therefore, split and/or inactivate any remaining ribosomes, functionally suppressing translation. This Goldilocks model of WhiB7 activity and aggregation rests on a key premise: translation rates should be decreased in WT, Δ*dosR* RV, and Δ*dosR*/Δ*whiB7* in aerobic stationary phase relative to Δ*dosR*. We therefore set out to directly test this hypothesis.

### Translation is necessary for aggregation/dispersal and translation rates correlate with re-aggregation

**Fig 4:**
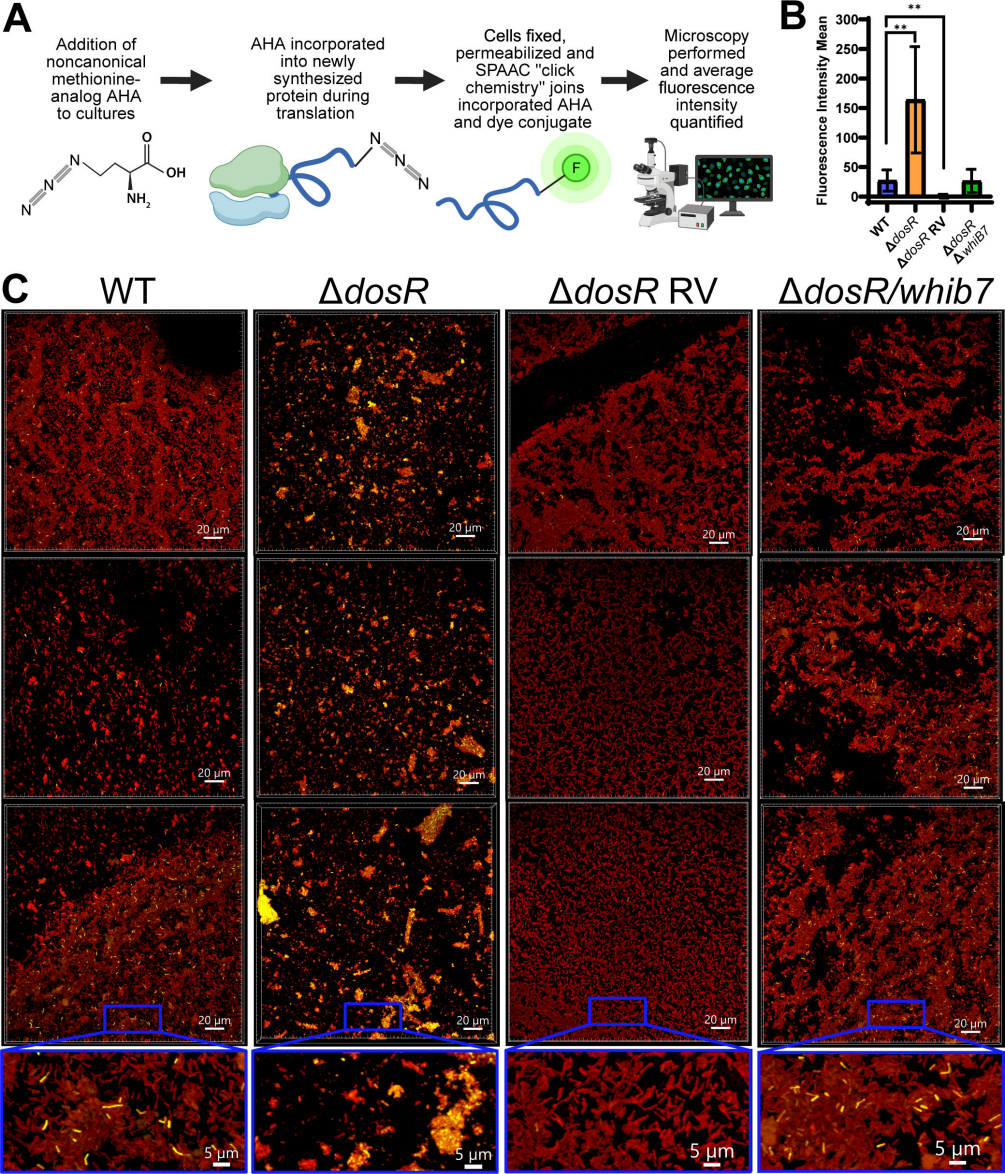
BONCAT of WT and Δ*dosR* mutants to quantify translation during late-stationary phase growth. (**A**) Bio-Orthogonal Non-Canonical Amino Acid Tagging (BONCAT) was performed in stationary phase (144 h) to measure translation in WT, Δ*dosR*, Δ*dosR* RV, Δ*dosR*/Δ*whiB7*. (**B**) Cells were stained with a BacLight red bacterial fluorescent stain, and the average fluorescence intensity of a Cy3-DBCO conjugate within a mask created from the BacLight channel was calculated for the AHA-treated cells. A corresponding H_2_O control (no AHA) was run for each strain. Each graph is the result of three replicate images taken from three biological replicates (nine total images per condition). The average fluorescence intensity of nine paired H_2_O control images was subtracted from each respective strain. The error bar is the standard deviation. (**C**) Three representative images of each strain. The average fluorescence intensity of the respective H_2_O controls was subtracted from the baseline fluorescence intensity of all voxels for each image. Panel **A** was created in Biorender. Meyer, M. (2026) https://BioRender.com/nvahqln

We utilized BONCAT to quantify the level of translation of WT, Δ*dosR,* Δ*dosR* RV, and Δ*dosR*/Δ*whiB7* strains during aerobic stationary phase (144 h). BONCAT allows for the quantification of translation occurring during a defined time period by introducing a non-canonical methionine analog azidohomoalanine (AHA), which is incorporated into newly synthesized protein (52, 53). Cells are then fixed, and the newly incorporated methionine analog is conjugated to a fluorophore (Cy3-DBCO in our case) by click chemistry (Fig. 4A) (52, 54). BONCAT samples were incubated with BacLight fluorescent stain, and fluorescence microscopy (Fig. 4C) was performed on three biological replicates of AHA-treated or control (no AHA treatment) WT, Δ*dosR*, Δ*dosR* RV, and Δ*dosR*/Δ*whiB7* in aerobic stationary phase (144 h). As predicted by our model, the Δ*dosR* mutant showed 6× higher signal than WT and the Δ*dosR*/Δ*whiB7* strains, which both showed a low level of translation consistent with slow growth during aerobic stationary phase (Fig. 4B). Also consistent with our model, the *whiB7*-overexpressing Δ*dosR* RV strain (Fig. 3B) showed a significantly lower level of translation than WT (Fig. 4B). Altogether, these results support our proposed model wherein *whiB7* activation in the Δ*dosR* mutant results in resumption of translation in aerobic stationary phase, allowing the cells to re-aggregate and re-disperse. However, the absence or overexpression of *whiB7* blocks translation in aerobic stationary phase, preventing aggregation.

### Exposure to reductive stress is sufficient to induce aerobic stationary phase re-aggregation in a *whiB7*-dependent manner

**Fig 5:**
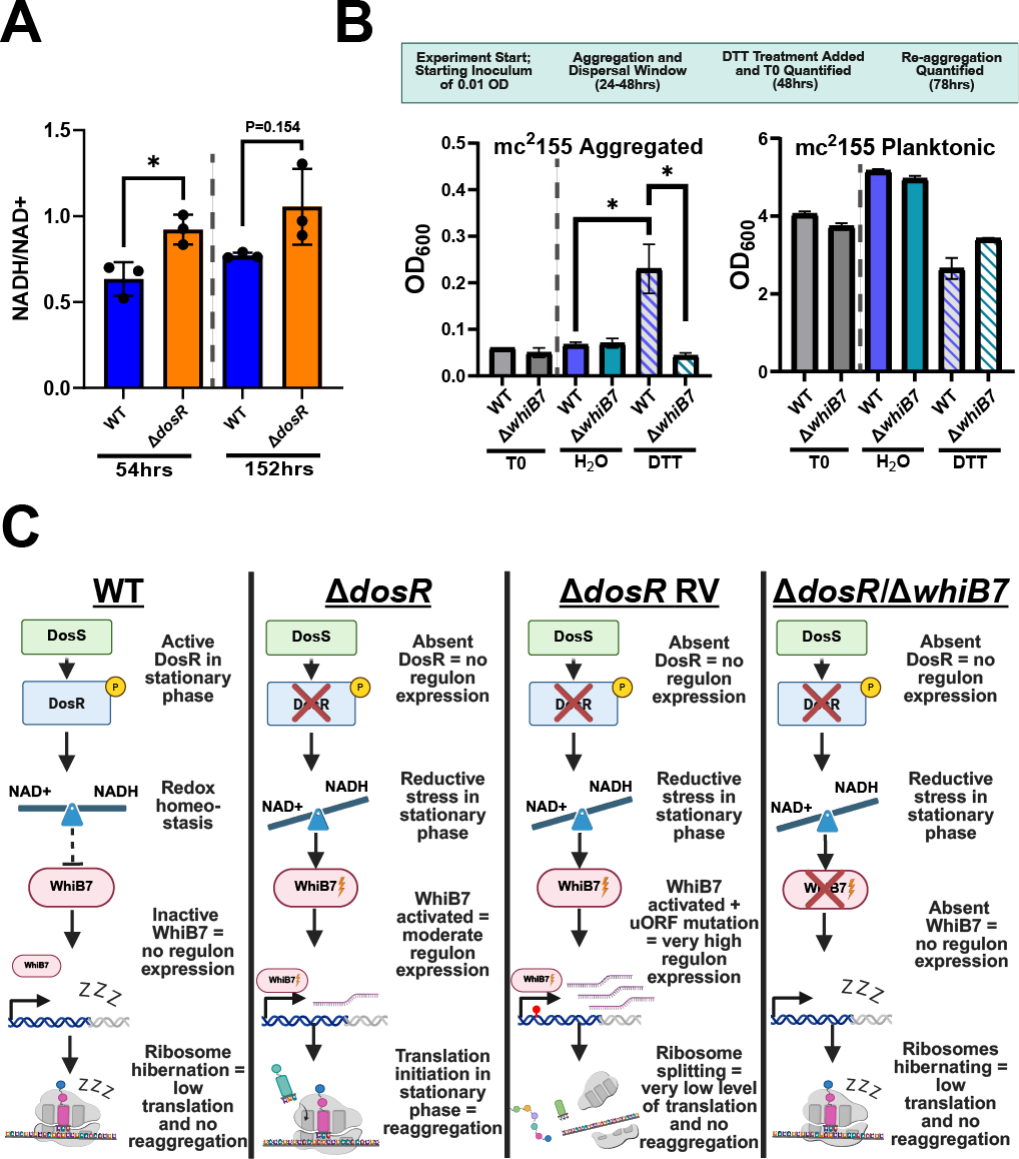
Reductive stress triggers *M. smegmatis* re-aggregation in a *whiB7*-dependent manner. (**A**) NADH/NAD+ ratio of WT and Δ*dosR* mutant in TYEM immediately following dispersal (54 h) and aerobic stationary phase (152 h). (**B**) WT and Δ*whiB7* mutant *M. smegmatis* were grown in TYEM until after dispersal (48 h). Cultures were harvested immediately (T0) or treated with 2 mM DTT (+DTT) or H_2_O and incubated for 30 h before harvesting (78 h). Each bar represents the average of three biological replicate cultures, with the error bars indicating the standard deviation. * represents a *P* value < 0.05. (**C**) Graphical model of DosR and WhiB7 regulation of translation during aerobic late stationary phase. In the Δ*dosR* mutant, reducing conditions sensitize WhiB7 leading to WhiB7-mediated ribosome rescue and resumption of translation. In the Δ*dosR* RV mutant, the overexpression of the WhiB7 regulon leads to high levels of ribosome splitting and subunit disorder, leading to decreased translational capacity. In the Δ*dosR*/Δ*whiB7* mutant, WhiB7 is not present to be sensitized and restart translation through ribosome rescue. Panels **B and C **were created in Biorender. Meyer, M. (2026), panel **B**, https://BioRender.com/kumnw2q; panel **C** https://BioRender.com/we5ncl5.

Our model and data suggest that WhiB7 activity is required for re-aggregation to occur in the Δ*dosR* mutant. We therefore hypothesized that an unknown signal in the Δ*dosR* mutant could be activating WhiB7 beyond its basal state during aerobic stationary phase growth. Reductive stress is a known activator of WhiB7 (38), and an *M. smegmatis* Δ*dosR* mutant has been shown to have a more reduced cytoplasm in comparison to WT under oxygen-limited stationary phase growth (28). To confirm that *M. smegmatis* Δ*dosR* also has a reduced cytoplasm in our aerobic stationary phase growth system, we directly measured the NADH/NAD+ ratio of WT *M. smegmatis* and the Δ*dosR* mutant. Indeed, compared to WT, Δ*dosR* had a more reduced cytoplasmic environment during early (54 h) and late stationary phase (152 h) (Fig. 5A). To directly test whether reductive stress could trigger WhiB7-dependent aggregation in WT *M. smegmatis*, we added the reducing agent DTT in aerobic stationary phase and found that it was, indeed, sufficient to trigger re-aggregation. Furthermore, DTT did not cause re-aggregation in Δ*whiB7*, suggesting that activating WhiB7 with reductive stress can drive aggregation in dormant cells even in the presence of a functional DosSR system (Fig. 5B). Δ*dosR* is sensitive to acidic stress during extended growth and may have increased acidification (55), but the addition of hydrochloric acid to a pH of 5.0 in aerobic stationary phase WT *M. smegmatis* cultures resulted in no change in aggregation (Fig. S4). Taken together, these results support and expand upon our model (Fig. 5C), suggesting that reductive stress generated in the Δ*dosR* mutant sensitizes WhiB7, triggering induction of the WhiB7 regulon, and inducing aggregation.

### WhiB7-activating antibiotics induce aggregation in both *M. smegmatis* and MABS clinical isolates

**Fig 6:**
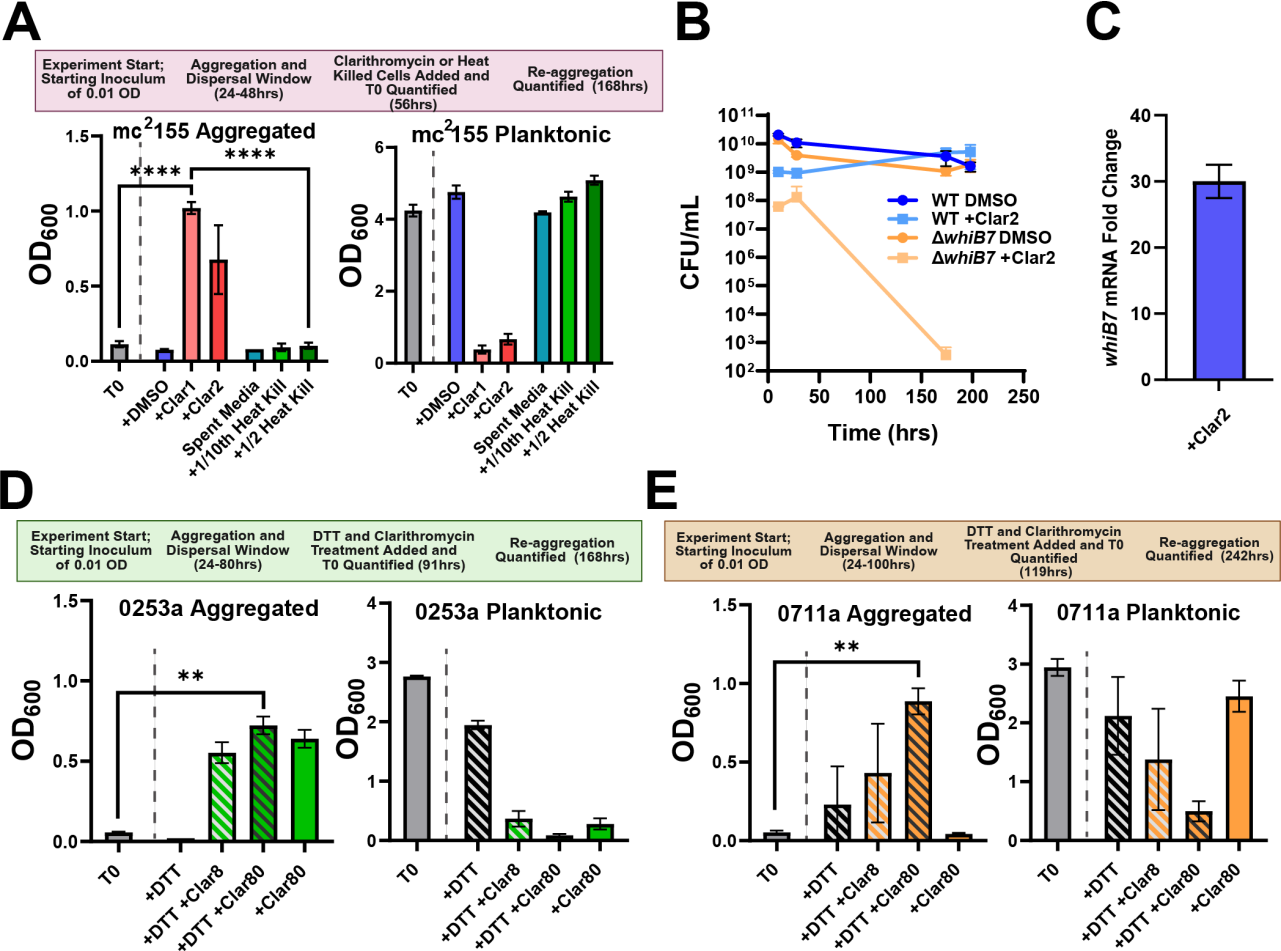
Treatment with WhiB7-activating clarithromycin can trigger stationary phase re-aggregation in both *M. smegmatis* and MABS. (**A**) WT *M. smegmatis* grown in TYEM until entrance into stationary phase (56 h). Cultures were harvested immediately (T0) or treated with 1 μg/mL or 2 μg/mL clarithromycin (+Clar1, +Clar2), DMSO (+DMSO), spent media, or 1/10th or 1/2 of a heat-killed (+1/10th Heat Kill and +1/2 Heat Kill) culture and incubated for 112 h before harvesting (168 h). 1 μg/mL or 2 μg/mL clarithromycin triggered re-aggregation in WT *M. smegmatis*. (**B**) CFU/mL of WT or Δ*whiB7 M. smegmatis* grown in TYEM before treatment with 2 μg/mL clarithromycin or DMSO control. (**C**) Fold change in *whiB7* mRNA in WT *M. smegmatis* after treatment with 2 μg/mL clarithromycin in comparison to DMSO control. After treatment during entrance to stationary phase (56 h), qPCR was performed on mRNA extracted from triplicate WT at 108 h. Expression levels were normalized against the housekeeping gene *mysA*, and WT mRNA after DMSO treatment was used as the non-experimental control. (**D**) MABS clinical isolate 0253a was grown in TYEM until entrance into stationary phase (91 h). Cultures were harvested immediately (T0), treated with 80 μg/mL clarithromycin (+Clar80), 1 mM DTT (+DTT), or a combination of 1 mM DTT and 8 μg/mL Clar (+DTT +Clar8) or 1 mM DTT and 80 μg/mL Clar (+DTT +Clar80) and incubated before harvesting at 168 h. (**E**) MABS clinical isolate 0711a with the same treatment with minor temporal adjustments to account for growth differences. Treatment was performed at 119 h, and samples were harvested at 242 h. Each bar represents the average of three technical replicate cultures, with the error bars indicating the standard deviation. * represents a *P* value < 0.05, ** a *P* value of <0.01, *** a *P* value of <0.005, and **** a *P* value of <0.0001. Panels **A, D, and E **were created in Biorender. Meyer, M. (2026), panel **A**, https://BioRender.com/puwie3y; panel **D**, https://BioRender.com/gwnoapd; panel **E**, https://BioRender.com/7sj54rs.

Our finding that DTT was sufficient to induce aerobic stationary phase aggregation in WT led us to hypothesize that other WhiB7-activating agents could do the same. Notably, specific classes of antibiotics which target the ribosome, including clarithromycin, a clinically relevant drug often used to combat MABS infections, can also activate WhiB7 (38). We therefore tested whether clarithromycin treatment alone, or in combination with sensitization from DTT, could trigger aggregation in stationary phase *M. smegmatis*. A subinhibitory concentration of clarithromycin (1 µg/mL, Table S3) was, indeed, able to trigger stationary phase *M. smegmatis* to re-aggregate (Fig. 6A). The Δ*whiB7* mutant was killed by clarithromycin exposure (Fig. 6B), so to test the involvement of WhiB7 in clarithromycin-induced re-aggregation, we performed qPCR. As expected, clarithromycin-treated cells showed 30-fold upregulated *whiB7* expression levels compared to DMSO control (Fig. 6C). Using a WT strain carrying FLAG-tagged WhiB7, we verified by western blotting that WhiB7 protein levels also increased after clarithromycin treatment (Fig. S5). Cell death can trigger biofilm formation in a variety of bacterial species, in some cases because the released peptidoglycan (56) or cytoplasm components like norspermidine (57) serve as a pro-biofilm signal to surviving cells. To test whether cell death was having an effect, we added heat-killed cells at a concentration of roughly 1 × 10^9^ (or 1/10th of culture cell count) and 5 × 10^9^ (or ½ of culture cell count) at the beginning of stationary phase. The addition of heat-killed cells did not induce aggregation, suggesting cell death was not causing the re-aggregation phenotype (Fig. 6A). Similarly, CFU counts of WT *M. smegmatis* after subinhibitory clarithromycin treatment showed similar survival to DMSO-treated cells, again suggesting cell death was not the principal driver of re-aggregation in our system (Fig. 6A and B). Altogether, these results indicate that sublethal concentrations of clarithromycin can trigger the activation of a WhiB7-mediated response which leads to aerobic stationary phase re-aggregation in *M. smegmatis*.

MABS responds to chemical cues of aggregation and dispersal in a similar manner to *M. smegmatis* (7, 58). We therefore tested whether smooth MABS CF isolate strains 0253a and 0711a would also re-aggregate in aerobic stationary phase in response to reductive stress and antibiotics. The strains showed unique responses to treatment, with 0253a re-aggregating in response to both near-MIC (Clar 8) and high dose (Clar 80) concentrations of clarithromycin (Table S3; Fig. S6) but demonstrating no sensitization by DTT (Fig. 6D). The 0711a strain was sensitized by DTT but did not respond to high clarithromycin on its own (Fig. 6E). Despite the heterogeneity, it was striking that all tested strains re-aggregated in aerobic stationary phase in response to antibiotic treatment.

### Hypoxic dormancy locks *M. smegmatis* into its aggregation state

**Fig 7:**
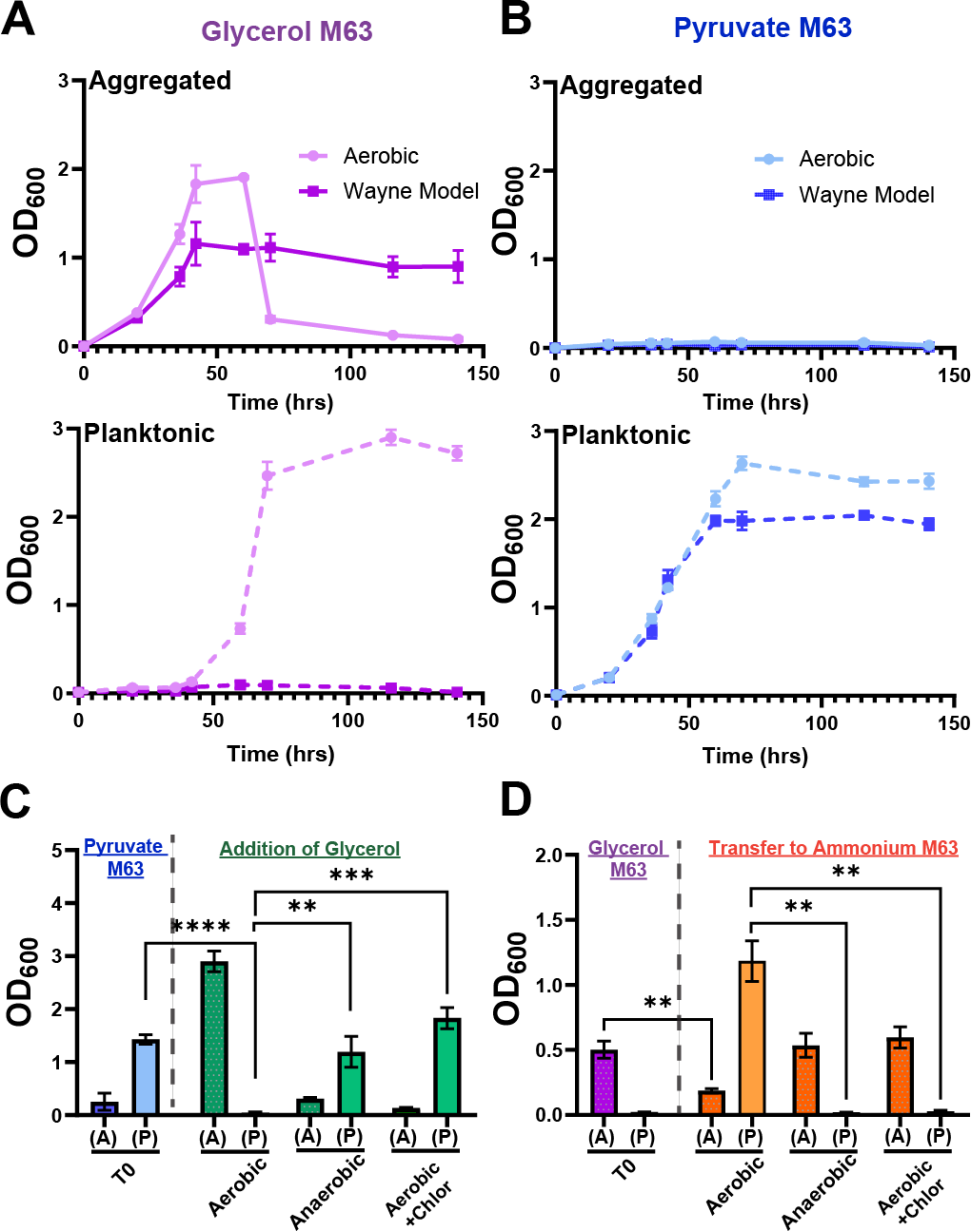
*M. smegmatis* is able to enter dormancy during both aggregate and planktonic growth. (**A**) Aggregation dynamics of *M. smegmatis* grown in Glycerol M63-1 under aerobic growth and using the Wayne model to induce hypoxic dormancy. (**B**) Aggregation of *M. smegmatis* in Pyruvate M63-1 under aerobic growth and using the Wayne model to induce hypoxic dormancy. Each timepoint represents the average OD_600_ values of the planktonic and aggregate fractions of three replicate cultures and the line indicates the standard deviation of the three replicates. (**C**) *M. smegmatis* grown in Pyruvate M63-2 under the Wayne model. At 42 h, cultures were spiked with glycerol to induce aggregation and harvested immediately (T0) or incubated for 8 h under aerobic, anaerobic, or aerobic + 25 µg/mL chloramphenicol conditions before harvest. (**D**) *M. smegmatis* grown in Glycerol M63-2 in the Wayne model. At 50 h, aggregates were harvested by filtration and resuspended in Pyruvate M63 + ammonia to encourage dispersal. Cultures were harvested immediately (T0) or incubated for 22 h in aerobic (standard culture tube growth), anaerobic (Balch tube growth), or aerobic + 25 µg/mL chloramphenicol, before harvesting and quantification. The average OD_600_ values of the planktonic “P” and aggregate “A” fractions of three replicate cultures are shown in each bar graph, and the line indicates the standard deviation of the three replicates. * represents a *P* value < 0.05, ** a *P* value of <0.01, *** a *P* value of <0.001, **** a *P* value of <0.0001.

Although it is a global dormancy regulator involved in the response to a variety of environmental stimuli, the DosSR system is classically and primarily considered through its involvement mediating the hypoxic dormancy response, permitting survival of mycobacteria in hypoxia (21, 22, 27, 59). Bacterial growth arrest derived from nutrient limitation differs from dormancy due to oxygen limitation in a few key ways, including differential availability of ATP (27, 31). To determine if aggregation dynamics were similar in different types of growth arrest, we tested whether the signals that drive re-aggregation in aerobic stationary phase would also permit re-aggregation under hypoxic dormancy. We first set out to test whether entrance into hypoxic dormancy itself affected aggregation or dispersal. For these experiments, we used defined M63 medium, which can be modified to support either planktonic or aggregated *M. smegmatis* growth (7). *M. smegmatis* cultured aerobically in Glycerol M63 grows predominately as aggregates (7). Here, we demonstrate that with longer incubations, these aggregates eventually disperse and grow planktonically (Fig. 7A). Aggregates grown in Glycerol M63-1 under the Wayne model entered dormancy without dispersing, suggesting that hypoxic dormancy does not drive dispersal (Fig. 7A). Similarly, Pyruvate M63-1 was used to grow planktonic cells, and under the Wayne model, they did not aggregate upon entrance into dormancy (Fig. 7B). These results indicate that *M. smegmatis* can enter hypoxic dormancy as both aggregates and planktonic cells.

Next, we tested whether hypoxic dormancy prevents *M. smegmatis* from responding to chemical aggregation or dispersal cues. The addition of glycerol to Pyruvate M63-2 grown planktonic cells did not induce aggregation in the Wayne model, but when these cells were aerated concurrently with glycerol exposure, they aggregated as expected (Fig. 7C). Similarly, aggregates grown in Glycerol M63-2 in the Wayne model did not disperse when resuspended in Ammonium M63 + Pyruvate, whereas dispersal occurred when the same cells were aerated (Fig. 7D). Taken together, these results indicate that entrance into hypoxic dormancy renders aggregates unresponsive to dispersal cues and can lock the cells into the state in which they entered dormancy. Consistent with our overall model (Fig. 5C), we also found that adding chloramphenicol, a translation-inhibiting antibiotic, to aerated cultures after chemical induction of aggregation or dispersal, inhibited both processes (Fig. 7C and D), providing evidence that aggregation and dispersal are both dependent on translation.

Lastly, we tested whether we could trigger re-aggregation in the Wayne model of hypoxic dormancy by stimulating WhiB7 with antibiotics. We grew *M. smegmatis* in diluted TYEM under the Wayne model and added clarithromycin after hypoxia-induced dormancy. We found that *M. smegmatis* did not aggregate in response to clarithromycin treatment in hypoxic dormancy. These results suggest core differences in the physiological state of *M. smegmatis* cells in aerobic stationary phase compared to cells under Wayne model hypoxia, whereby only stationary phase induced slow growth is permissive to WhiB7-mediated re-aggregation (Fig. S7).

## DISCUSSION

Many environmental reservoirs that NTM inhabit, such as water distribution systems and the rhizosphere, have fluctuating temperatures, humidity, and nutrients. Growth in these environments can also expose NTM to bacteriostatic and bactericidal agents produced by competing bacteria and other antimicrobial chemicals like chlorination (60–62). Some NTM, including MABS, are opportunistic pathogens that can cause chronic pulmonary infections, especially in people with underlying lung conditions such as cystic fibrosis (CF), and the infection environment is not any friendlier (63–67). In addition to exposure to the host immune response, large regions of CF sputum and other chronic infection environments are hypoxic, as evidenced by direct measurements of CF sputum and an enrichment of hypoxia-induced *P. aeruginosa* transcripts in sputum samples, and mycobacteria require oxygen to replicate (65, 68). Nutritional immunity by the host can also limit essential nutrients in the infection environment, causing nutrients to fluctuate dramatically, and antibiotics are ever-present (63–65, 69, 70). Direct measurements of *Staphylococcus aureus* growth rate *in situ* support a limiting nutritional environment, demonstrating slow growth during CF pulmonary infections (2, 64, 65). Since NTM forms biofilms in both host and non-host environments (16, 71, 72), it seems highly likely that they encounter nutrient-limited environments which would halt growth and limit metabolic activity of the entire biofilm community, thereby creating metabolically dormant aggregates. In order to develop agents which effectively trigger NTM biofilm dispersal, we therefore must understand how dormancy affects entrance and exit from the biofilm state. Thus, we designed this study to elucidate how the regulatory pathways controlling aggregation and dispersal function under dormancy.

Our results suggest that in aerobic stationary phase, the activation of the WhiB7 system can allow for aggregation, at least in part, by restarting active translation at moderate/optimal expression levels. Known activators of the WhiB7 system, such as treatment with the reducing agents BME and DTT and the WhiB7-triggering antibiotic clarithromycin, permitted *M. smegmatis* aggregation in aerobic stationary phase growth. Our data and the literature suggest that multiple WhiB7-regulated components may be involved in restoring translation and permitting aggregation when expressed at optimal levels, but these factors may impair translation when over-expressed, such as in the Δ*dosR* RV mutant. The WhiB7 regulon includes the ribosome-splitting factor *hflX* (42–45). HflX is a GTPase that associates with stalled ribosomes, including ribosomes targeted by antibiotics like macrolides, and splits the stalled 70S ribosomes by disordering and sequestering the 50S subunit, freeing the mRNA, and allowing for translation to resume when antibiotic concentrations decrease due to *erm* regulated responses and/or metabolic conditions improve (42, 43). However, it has also been shown that HflX binds to antibiotic-free and active ribosomes (43), and it is hypothesized that this can cause them to excessively dissociate as evidenced by the overexpression of *hflX* from a strong promoter on a multicopy plasmid having a lethal effect in *M. smegmatis* (42). It stands to reason that the overexpression of the WhiB7 regulon could lead to excess HflX production, and the associated increase in HflX-mediated ribosome splitting and 23S disorder could reduce the translational capacity of the cell. In addition to HflX, WhiB7 also regulates *erm(38)* (46), the *M. smegmatis* homolog of *erm*, the highly conserved inducible macrolide resistance factor in NTM (47, 48, 51). These 23S rRNA methyltransferases add one or two methyl groups to the macrolide binding site preventing the drug from binding and increasing the macrolide resistance of the cell (47, 48, 51). However, as has been seen in another *erm* containing bacterium, *Staphylococcus aureus* (50), these *erm*-methylated ribosomes can have decreased translation efficiency and an altered translational profile leading to increase fitness cost in antibiotic-free growth (51). Altogether, these data indicate that multiple WhiB7-regulated factors could play a role in decreasing translation in our *whiB7*-overexpressing Δ*dosR* RV mutant.

WhiB7 activation in response to antibiotics, reductive stress, or amino-acid depletion relies on two levels of regulation. First, transcription of *whiB7* is controlled by a rho-independent terminator prior to the *whiB7* ORF, which under normal uORF translation leads to early termination prior to *whiB7* ORF transcription (41, 73). Under conditions in which ribosome stalling occurs during translation of the uORF, like exposure to ribosome-targeting antibiotics or amino acid depletion, anti-terminator formation allows for transcriptional readthrough and expression of the *whiB7* ORF (73). There is also direct upregulation of promoter activity after anti-terminator formation (73). The WhiB7 protein is then sensitized by reducing conditions and upregulates the expression of itself in a feed-forward manner (38, 41, 46). Our results suggest that reductive stress in the Δ*dosR* mutant could trigger WhiB7 activity and allow for increased translation to take place. The lack of aggregation in our Δ*dosR*/Δ*whiB7* double mutant indicates that WhiB7 activity is essential for aerobic stationary phase aggregation, but our finding that *whiB7* transcripts were not markedly upregulated in the Δ*dosR* mutant compared to WT suggests there may be more subtle temporal dynamics at play. In *Mycobacterium tuberculosis*, a fivefold increase in *whiB7* expression is observed at the start of aerobic stationary phase, but expression falls to a basal level in late aerobic stationary phase (74). Our qPCR was performed at 170 h, late aerobic stationary phase for *M. smegmatis*, so it is likely that the transcriptional response in Δ*dosR* may have leveled off by that point. In the Δ*dosR* mutant, our model predicts that the WhiB7 that was produced in early aerobic stationary phase is sensitized by the reduction in cytoplasmic redox state and activates its regulon enough to permit re-aggregation. In contrast, the extreme induction of *whiB7* in Δ*dosR* RV mutant, even in late aerobic stationary phase, and the ablation of new translation suggest that very high WhiB7 activity can inhibit new translation during that growth phase. Alternatively, other components of the WhiB7 regulon may also affect the lack of translation in the Δ*dosR* RV mutant. For example, a WhiB7-mediated increase in intracellular pools of mycothiol, an important redox-balancing thiol buffer similar to glutathione, may ultimately rebalance intracellular redox and could suppress WhiB7 activation (38). There may also be involvement of additional transcription factors, such as the intracellular redox sensor WhiB3, which plays a role in the response to reductive stress and hypoxia (75). These possibilities merit further consideration and will need to be explored in future studies.

The very high level of translation in the Δ*dosR* mutant in comparison to WT during aerobic stationary phase indicates that the absence of *dosR* may sensitize the cell to WhiB7 activity. The reductive stress of Δ*dosR* causing constitutive activation of WhiB7 could account for this. Alternatively, in aerobic stationary phase, *M. smegmatis* demonstrates an accumulation of inactive large ribosomal subunits (LSUs) and ribosomes associated with HPFs, including Balon homolog MSMEG_1130, RafS/MPY (MSMEG_1878), and RafH (MSMEG_3935) which can bind to stationary phase ribosomes and inhibit protein synthesis (29, 76–78). In the Δ*dosR* mutant, due to the absence of DosR-mediated induction of HPF expression, there could be a larger population of stalled, WhiB7-susceptible ribosomes rather than hibernating ribosomes bound to HPFs in aerobic stationary phase. If WhiB7-mediated ribosome splitting is necessary for active translation to take place, that may explain the very high translation in Δ*dosR* (Fig. 4B). This is supported by the comparatively low level of aggregative response of WT *M. smegmatis* after treatment with DTT (Fig. 5B) in comparison to the dramatic re-aggregation seen in the Δ*dosR* mutant during aerobic stationary phase (Fig. 1D, 2B and 3C). Differential expression and activity of these HPFs under Wayne model hypoxia and aerobic stationary phase growth may also help explain why the aerobic stationary phase cells were susceptible to induction of aggregation, while the hypoxic dormant cells were not (76, 78). In total, our data provide evidence for a Goldilocks-type regulation of aggregation, wherein WhiB7-mediated re-activation of translation is necessary to allow for the production of a pro-aggregation signal, but too much *whiB7* expression can prevent translation through excessive ribosome splitting and inactivation.

The identification of the specific proteins that are translated in a WhiB7-dependent manner and drive aggregation will be the focus of future work. Various pieces of data from this study and others may provide hints. For example, while the Δ*dosR* mutant had decreased aggregation during the initial aggregation and dispersal window in exponential growth (Fig. 1E, 2C and 3D), WT levels of aggregation were restored in the Δ*dosR*/Δ*whiB7* double mutant. This indicates that DosSR and WhiB7 activity also impact aggregation in active growth, albeit in seemingly opposite directions compared to in aerobic stationary phase. In addition to controlling global translation, DosR and WhiB7 could also transcriptionally control potential aggregation factors or influence the pools of biosynthetic precursors of aggregation factors. Previous studies have shown that the treatment of *M. tuberculosis* (79) and NTM, including MABS (80), with high levels of reductive stress (6 mM DTT) leads to the formation of cellulose-rich pellicle biofilms which are extremely hydrophobic and resistant to dispersal. However, another study performed with a MABS CF clinical isolate strain in synthetic cystic fibrosis medium, showed no increase in biofilm formation after treatment with 6 mM DTT, and saw that these biofilms were not responsive to cellulase treatment, indicating that there may be a difference in cellulose and eDNA composition between *M. tuberculosis* and NTM biofilms (81). Here, we see that lower concentrations of DTT treatment (2 mM for *M. smegmatis*) triggered aerobic stationary phase biofilm formation in WhiB7-dependent manner, and the WhiB7-activating antibiotic clarithromycin was also able to trigger this effect. The genes required for mycobacterial cellulose or eDNA production have not been well described, and WhiB7 has not been previously linked to cellulose or eDNA production, but these components are still potential candidates for the WhiB7-dependent aggregation factor. Additionally, previous studies in MABS determined that the genes encoding hypothetical protein MAB_3786c, and the lipoprotein LppF, MAB_3785c, are among the most downregulated genes in a Δ*whiB7* MABS mutant (44). Lipoproteins have various roles in mycobacteria, including acting as adhesins and regulating cell wall lipid transport and biosynthesis. *M. smegmatis lpqW* (MSMEG_5130) is a highly conserved gene that has been seen to play a role in the conversion of phosphatidylinositol mannosides (PIMS) to lipoarabinomannans (LAM), important mycomembrane components (82). Future work will aim to clarify the pathway from WhiB7 activation to aggregation. The DosR regulon also controls putative adhesins. For example, one of the highest upregulated genes in the DosR regulon upon entrance into hypoxia is the triacylglycerol (TAG) synthase *tgs1* (83), which is the major enzyme involved in TAG biosynthesis. TAG is accumulated within intra bacterial inclusions (ILI), which are critical for survival under hypoxia and for rapid recovery from dormancy (18). TAG biosynthesis has also been conversely tied to the production of mycolyl-diacylglycerols; hydrophobic apolar lipids made up of a mycolic acid esterified to a glycerol that increase cell membrane hydrophobicity and play a role in biofilm formation (84).

Bacterial aggregation can be energy-dependent, requiring transcription, translation, and matrix production. However, in some circumstances, such as *P. aeruginosa* undergoing spontaneous depletion aggregation triggered by an increased concentration of non-adsorbing polymers in the culture growth media, aggregation does not require any of those processes (85). Previously, we have shown that NTM aggregate and disperse in response to carbon and nitrogen availability and that this aggregation is an essential early step in colony and pellicle biofilm development (7, 9). However, we had not empirically established that NTM aggregation and dispersal are active processes requiring cellular energy expenditure. Our new finding that chloramphenicol blocks aggregation and dispersal indicates that entrance and exit from NTM aggregated growth are dependent upon translation, implying it is a regulated bacterial response to environmental stimuli that requires energy. Indeed, we assert that control of translation and the translational capacity of the cell appear to serve as regulatory checkpoints controlling aggregation. In hypoxic dormancy, we found that *M. smegmatis* did not aggregate or disperse in response to nutrient cues or in WhiB7-stimulating antibiotics. It may be that oxygen starvation leads to a harsher restriction on translation due entirely to energetic constraints, or it may be that, although DosSR is active in both conditions, there are regulatory layers present in oxygen restriction that do not allow aggregation to occur. Indeed, hypoxia results in stronger induction of the DosSR regulon than aerobic stationary phase, suggesting there could be a regulatory role (21). Future studies will work to distinguish between these two possibilities.

Altogether, our results support a model wherein planktonic, aerobic stationary phase NTM can aggregate when WhiB7 is triggered, and this re-aggregation is dependent upon new protein translation. Importantly, because our re-aggregated cells also re-disperse (Fig. 1D, 2B and 3C), our data suggest that chemical dispersal of certain dormant NTM biofilms is possible. Targeting the systems that trigger dispersal under hypoxic conditions may not be effective, potentially because these systems require energy or oxygen that the cell does not have. Future studies defining the specific mechanism by which hypoxia prevents NTM aggregation and dispersal may help determine how best to wake up dormant biofilms for dispersal and open up new avenues of therapeutic design. Additionally, this study led us to an unexpected and very clinically relevant phenomenon, the antibiotic clarithromycin, an established drug of choice for MABS infections, is sufficient to trigger aggregation of *M. smegmatis* and MABS clinical isolates when administered to dormant (but oxygenated) cells in late aerobic stationary phase. This effect has precedence as sub-inhibitory concentrations of antibiotics have been seen to stimulate biofilm formation in other species like *Escherichia coli* and *P. aeruginosa* (86, 87). Since MABS biofilm formation is well documented to confer antibiotic tolerance, and the tolerance derived from WhiB7 activation of the *erm(41)* system has been seen to confer broad spectrum tolerance of antibiotics (44, 46, 48), these results re-iterate the importance of considering how treatments may inadvertently trigger WhiB7. In line with this concern, a recent study has shown that the treatment of MABS with tobramycin (which is commonly used to treat *P. aeruginosa* infections) increased *whiB7* expression and that this increased tolerance of oxidative stress and increased survival in human macrophages (88). DosSR has also been a frequent target of interest for drug development because the activation of the system results in the formation of non-replicating cells which are difficult to treat (89, 90). However, our study suggests that the inhibition of the DosSR system may sensitize the WhiB7 response, reactivating translation and triggering biofilm formation. Pairing an anti-DosR treatment with an adjuvant which helps to maintain redox homeostasis may allow for the inhibition of DosR-mediated hypoxic dormancy without sensitization of WhiB7. Our findings give credence to the idea that the re-activation of translation in dormant biofilm will be critical to inducing dispersal, and they highlight the need to avoid triggering intrinsic resistance systems like WhiB7 when designing these activation factors. More broadly, these results increase our understanding of the interactions between two systems critical to survival of adverse environmental conditions, entrance into dormancy and biofilm formation.

## MATERIALS AND METHODS

### Bacterial strains and growth conditions

The strains, plasmids, and primers used in this study are summarized in Table S1. For general culturing, mycobacteria were grown in TYEM (10 g/L tryptone, 5 g/L yeast extract, 2 mM MgSO4) in borosilicate glass tubes, incubated at 37°C with 250 rpm shaking. For the Wayne model experiments performed in this study, a modified M63 medium was used with the following base concentrations (100 mM KH2PO4, 0.5 mM proline, 1 mM MgSO4, 10 µM FeSO4, 1× SL-10 trace metal solution, filter sterilized), and carbon sources were added as follows: Glycerol M63-1 (30 mM glycerol 5 mM sodium glutamate), Glycerol M63-2 (60 mM glycerol 10 mM sodium glutamate), Pyruvate M63-1 (30 mM sodium pyruvate, 5 mM sodium glutamate), Pyruvate M63-2 (30 mM sodium pyruvate, 30 mM sodium glutamate), Ammonium M63 (60 mM NH4Cl), Ammonium M63 + Pyruvate (60 mM NH4Cl, 10 mM sodium pyruvate).

To create a Δ*dosR* mutant in *M. smegmatis* mc^2^155, recombineering was used according to the established protocol (7). This method was used to make the Δ*dosR*/Δ*whiB7*, and Δ*whiB7* mutants with the minor modifications of a 30 min incubation of ice after glycerol washes, the use of a hygR resistance cassette for selection, and 800 ng linear DNA was used during electroporation. Mutants were verified by PCR to confirm gene replacement and cloning junctions were verified by Sanger sequencing. As pJV53 and our complement vector pMH94 have the same kanamycin selection, for complementation of Δ*dosR*, hygromycin selected plasmid pMH94.hyg was created from the pMH94 backbone by NEBuilder HiFI DNA Assembly (NEB E2621L). Plasmids were verified by full plasmid sequencing and transformed into electrocompetent *M. smegmatis* with the modifications to the electro-transformation method which are described above.

Whole-genome extractions were performed as previously described on WT *M. smegmatis* mc^2^155, the Δ*dosR*, and Δ*dosR* RV mutants (91). Genome samples were then sent out for Oxford-Nanopore and Illumina whole-genome sequencing. A Breseq mutant analysis pipeline was used to identify mutations which had emerged in the Δ*dosR* RV in comparison to the ΔdosR mutant. After trimming synonymous mutations and excluding any duplicate mutations found in both Δ*dosR* and Δ*dosR* RV, the remaining non-synonymous mutation is a G->A mutation 390 bp upstream of *whiB7* and within the uORF region of *whiB7* (MSMEG_1953).

### Aggregation assays

Aggregation assays were performed with the separation of aggregate and planktonic cell population by passage over a 10 µm strainer as previously described with minor modifications (7). Briefly, as previously described (7, 9), TYEM was inoculated to an OD_600_ of 0.01 with the strain of interest, and 5 mL aliquots were dispersed into replicate culture tubes and grown at 37°C shaking at 250 rpm. Then at intervals throughout culture maturity, three tubes per condition were removed and the aggregate and planktonic fractions were separated by passing over a 10 µm strainer which retained the aggregated cells. For this study, to increase efficiency dispersing aggregates, rather than 500 µL of Tween 20, 500 µL of Tween 80 was added to the tube before the aggregates were resuspended by vortexing and sonication and the OD_600_ was recorded. The Δ*dosR* planktonic OD_600_ not returning to WT levels after re-dispersal is likely due to the aggregates not fully dispersing by the final timepoint and the loss of planktonic cells transiently attached to aggregates or small aggregates caught on the 10 µm filter, which are lost during the PBS wash of the filter-attached aggregates.

For the determination of the nutrient limitation driving stationary phase growth arrest in TYEM (Fig. S2), cells were grown in TYEM supplemented with the indicated chemical. The pH of the MOPS supplemented TYEM was adjusted to 7.2 using KOH pellets. Cultures were harvested at stationary phase growth (108 h), and the pH of triplicate cultures was recorded using a Fisherbrand accumet AB250 pH meter (13-636-AB250A).

### Balch tube aggregation assays

Dormant populations of cells were generated following a modified Wayne model. Briefly, 5 mL of the indicated M63 medium was inoculated to an OD_600_ of 0.01 and dispensed into sterile Balch tubes, crimped closed, and grown shaking at 37°C and 250 rpm. This allows for the gradual creation of a hypoxic environment as the headspace oxygen is depleted. In parallel with the Balch tubes, 5 mL aliquots of the same inoculated M63 were dispensed into standard disposable borosilicate tubes and grown aerobically. Quantification of aggregation was performed using the strainer aggregation assay.

To compare the ability of aggregated cells to disperse under aerobic growth or hypoxic dormancy (Fig. 7A), Glycerol M63-1 was used, as the lower concentration of glycerol and glutamate resulted in faster and more efficient dispersal under aerobic conditions, due to faster depletion of carbon (Fig. S2A), allowing for comparison against the Balch tube grown. However, M63 Glycerol-2 was used for the Balch tube induction of dispersal (Fig. 7D) to guarantee the aggregated cells were carbon replete at time of harvest as to mitigate the role of dormancy due to carbon depletion, and residual glycerol and glutamate were removed during the rinsing of the aggregates and resuspension in Ammonium M63.

Similarly, to compare the growth of planktonic cells under aerobic growth and Wayne model, planktonic cells were grown in Pyruvate M63-1, which has a low level of glutamate, promoting growth exclusively as planktonic cells. However, for the Balch tube induction of aggregation experiments, Pyruvate M63-2, which has a higher concentration of glutamate, was used to allow for easier recovery from dormancy and allow a rapid transition to aggregated growth when transferred from Balch tube to aerobic growth.

For the Balch tube aggregation induction experiments (Fig. 7C), replicate tubes of mc^2^155 were grown in Pyruvate M63-2 (5 mL) in Balch tubes shaking at 37°C and 250 rpm. Tubes were grown into the start of hypoxic dormancy (50 h) at which time three tubes were immediately harvested and quantified to provide a T0 measurement of aggregation. The remaining nine tubes were then subjected to one of three conditions in triplicate: (i) 900 µL of vacuum-degassed sterile 1 M glycerol (180 mM final concentration) added to the Balch tube with a needle through the septum; (ii) transferred to a borosilicate culture tube by decanting and then proceeding to add glycerol; (iii) transferred to a borosilicate culture tube and adding 25 µg/mL chloramphenicol along with the glycerol. The tubes were grown for an additional 10 h, and then the aggregation assay was performed.

For the Balch tube dispersal induction experiments (Fig. 7D), replicate tubes of mc^2^155 were grown in Glycerol M63-2 (5 mL) in Balch tubes shaking at 37°C and 250 rpm. Tubes were grown into the start of hypoxic dormancy (42 h), and aggregates were harvested by filtration under vacuum in an anaerobic chamber. The aggregates were rinsed with Ammonium M63, before being resuspended in Ammonium M63 + Pyruvate and dispensed into nine borosilicate tubes for aerobic growth and three Balch tubes which were sealed for anaerobic growth. The tubes were removed from vacuum, and three standard tubes were immediately harvested in order to provide the T0 measurement of aggregation. Three standard aerobic tubes were treated with 25 µg/mL chloramphenicol, and three tubes were grown aerobically without antibiotic. Tubes were incubated for additional 22 h before harvesting and quantifying aggregation.

For the Balch tube clarithromycin experiments (Fig. S7), replicate tubes of mc^2^155 were grown in 2:1 diluted TYEM (5 mL) rather than M63 defined media in order to generate fully planktonic cells under hypoxia in nutritional environment more similar to the TYEM in aerobic late stationary phase. Cultures were grown shaking at 37°C and 250 rpm. Tubes were grown into hypoxic dormancy (56 h) when cultures were harvested immediately (T0) or treated with 2 µg/mL clarithromycin or DMSO control and incubated for 132 h under anaerobic conditions before harvest.

### CFU counts

CFU counts were obtained by the drip plate method as previously described with minor modification (7). Briefly, 100 µL of sterile Tween 20 was added to cultures before they were vortexed and water bath sonicated until no visible aggregates remained. One hundred microliters of the suspended cell culture was removed, and serial dilutions spanning seven orders of magnitude were performed. Ten microliters of each dilution was plated onto TYEM agar plates, and the plate was tilted to spread out each drip. Plates were incubated at 37°C until colonies were formed (2–3 days for *M smegmatis*, 3–4 days for *M. abscessus*). Colonies were counted at the appropriate dilution and reported CFUs/mL are the average of three biological replicates.

For the survival of MABS CF isolates after clarithromycin treatment (Fig. S6), MABS isolate 0253a and 0711a cultures were grown in TYEM (5 mL) until after dispersal at 91 h. CFUs were plated to establish baseline, and cultures received treatment with 8 or 80 µg/mL clarithromycin or a DMSO control. Cultures were incubated at 37°C with 250 rpm shaking before CFUs were taken at 24 h, 72 h, 168 h post treatment (115 h, 163 h, 259 h after inoculation). Drip plating was performed as above.

### Colony morphology imaging

For comparison of colony morphology between WT *M. smegmatis* and mutant strains, each strain was grown in TYEM at 37°C with 250 rpm shaking. The cultures were then normalized to an OD_600_ of 1 before 4 µL of WT, and each mutant was spotted onto TYEM agar plates, incubated for 5 days at 37°C, and imaged using a Zeiss Stereo Discovery V8 microscope.

### NADH/NAD+ measurement

NADH and NAD+ extraction was performed as previously (92) with the modification that 2 mL of cell culture was used as the basis for extraction; the suggested lower volume (200 µL) of NaOH and HCl was added to the cell pellets, but only 5 µL of extract was analyzed rather than 15 µL.

### qPCR

RNA extraction for comparison is of WT, and Δ*dosR* mutant qPCR (Fig. 3B) was performed as previously (38) with some modifications. The initial starting cultures of WT mc^2^155 and the Δ*dosR* mutants were at late stationary phase (160 h) rather than cultures at OD_600_ of 0.6–0.8. Each of three replicates was composed of two 5 mL cultures combined in a 15 mL Falcon tube and pelleted by centrifuging at 5,000 *g* for 10 min at 4°C. The supernatant was decanted, and 5 mL of GTC Buffer was used to resuspend and transfer the pellet to 35 mL GTC Buffer, resulting in a final GTC Buffer ratio of 4:1 to initial culture volume. One milliliter of TriZol was used instead of 1 mL of QiaZol and Precellys Tough Micro-organism Lysing kit VK05 (Bertin Corp Product # P000913-LYSK1-A.0), and an Omni Bead Ruptor 12 was used for the bead beating (at 4°C, 6 m/s speed, 3 cycles, 45 s per cycle, 5 min break on ice between cycles). The lysate was transferred to a 1.5 mL microcentrifuge tube rather than a phase-lock and incubated at RT for 5 min, and 200 µL of ice-cold chloroform was added. The contents were immediately mixed by inversion for 30 s, incubated at RT for 5 min, and centrifuged at 4°C for 5 min at 16,000 *g*. After initial Turbo DNase (Ambion, Cat # AM2238) treatment, two subsequent rounds of DNAse treatment were performed according to manufacturer’s instructions, with a Purelink RNA mini kit (Ambion, Cat # 12183018A) spin column cleanup between treatments. The RNA was eluted in RNAse-free H2O and quantified using a Qubit 4 Fluorometer (Invitrogen, Q33238).

cDNA synthesis was performed using the iScript cDNA synthesis kit (BIO-RAD, 1708890) according to the protocol given by the manufacturer. For the WT vs Δ*dosR* mutant qPCR, 1 ng of the purified RNA used as template, and for the WT *M. smegmatis* after clarithromycin treatment, 100 ng was used.

Quantification of the relative abundance of *whiB7* RNA was performed using the PowerUp SYBR Green Master mix (Applied Biosystems, A25742) and QuantStudio3 real-time PCR system (Applied Biosystems, A28137). No reverse transcriptase control was performed in parallel to quantify any contaminant DNA in the RNA samples. For WT vs Δ*dosR* mutant qPCR, WT relative abundance was used as the control. mRNA relative abundance was determined using the house-keeping gene mysA, and the relative abundance of *whiB7* mRNA of each mutant was compared against the mRNA level seen in WT. For clarithromycin-treated WT cells, *mysA* was used as the housekeeping gene, and expression levels of clarithromycin treated cells were compared against DMSO-treated control. PCR-efficiency test was used to compare primer sets, and the given Ct values were adjusted accordingly. ΔΔCt analysis was performed (93), the results of which are seen in Table S2.

For *whiB7* activation after clarithromycin (Fig. 6C), cells were treated with 2 µg/mL clarithromycin or DMSO control at 56 h and flash frozen at 140 h. RNA extraction was performed from these freezer stocks of cells using the instruction from the Purelink mini kit, “Purifying RNA from Bacterial Cells with On-column Purelink DNase Treatment” with the following changes; Step 6, homogenization of the cell lysate, was performed using Precellys Tough Micro-organism Lysing kit VK05 bead tubes (Bertin Corp Product # P000913-LYSK1-A.0), and Omni Internation Bead Ruptor 12 machine was used for the bead beating (at 4°C, 6 m/s speed, 3 cycles, 45 s per cycle, 5 min break on ice between cycles); all other steps were performed as described. After elution with 50 µL RNase-free H2O, 5 µL of RNA was set aside, and the remaining 45 µL was digested with Turbo DNase according to manufacturer’s instructions, with a Purelink RNA spin column cleanup after treatment. Purity of RNA samples was assessed by Nanodrop OneC (Thermo Scientific, Cat #: ND-ONE-W). cDNA synthesis, RT-PCR, and ΔΔCt analysis were performed as outlined above.

### Western blot analysis

ORBIT cloning was performed as previously described (94) to create *M. smegmatis* strains harboring a C-terminal EGFP-4xGly-TEV-Flag-6xHis tag on *whiB7* (MSMEG_1953). The tagged strain, and WT *M. smegmatis,* was grown until after dispersal (56 h) in TYEM at 37°C with 250 rpm shaking. Strains received treatment with 2 µg/mL clarithromycin or DMSO and were incubated for 76 h (132 h post inoculation), before lysis as previously described (95). Ten microliters of sample was loaded onto 4%–15% Mini-Protean TGX protein gels (BioRad #4561084) and was run in 25 mM Tris, 192 mM glycine, 0.1% SDS, pH 8.3 running buffer at 150 V for 35 min. Gels were transferred to PVDF membrane using 25 mM Tris, 192 mM glycine, 20% methanol transfer buffer with an icepack, at 100 V for 1 h. Membranes were blocked at 4°C overnight in 1× PBS, 0.1% Tween 20, 7% BSA. The primary antibody incubation was for 2 h at RT in 1 µg/mL mouse anti-FLAG M2 antibody (Sigma-Aldrich) in 1× PBS, 0.1% Tween 20, 1% BSA, 0.1% sodium azide. The secondary antibody incubation was for 1 h in 1:10,000 horseradish peroxidase-coupled goat anti-mouse IgG antibody in 1× PBS, 0.1% Tween 20, 1% BSA. Five washes at RT for 5 min in 1× PBS, 0.1% Tween 20 were performed after blocking, primary antibody, and secondary antibody incubations.

### BONCAT labeling and fluorescence microscopy

#### AHA labeling

Azidohomoalanine (AHA) was purchased from Vector Laboratories (SKU: CCT-1066). Cultures were grown in TYEM until late stationary phase (144 h), and 100 µL of sterile 50 mM AHA (1 mM) or H_2_O control was added to triplicate cultures. The cells were then incubated for 3 h at 37°C with 250 rpm shaking to allow labeling to occur. Cells were then pelleted and washed three times with sterile 0.9% NaCl to remove residual AHA, fixed by incubation with 4% paraformaldehyde (PFA) at RT for 30 min, and washed another three times with 0.9% NaCl to remove residual PFA.

#### Click chemistry

Fixed cells were resuspended in 200 µL ice-cold 70% ethanol and incubated on ice for 1 h. Cells were rinsed with 0.9% NaCl, before being resuspended in 200 µL 46°C pre-warmed 100 mM iodoacetamide in 0.9% NaCl. The cells were incubated at 46°C for 1 h covered from light, pelleted, and then washed with 0.9% NaCl. Cells were then pelleted and resuspended in 100 µL 20 µM Cy3-DBCO (CCT-A140 from Vector Laboratories) in 0.9% NaCl followed by a 30 min incubation at RT in the dark. Cells were then pelleted again, washed three times with 0.9% NaCl, and resuspended in 20–800 µL H2O according to pellet size.

#### Microscopy

One microliter of each BONCAT sample was heat fixed to a glass microscopy slide and covered in 1 µL of 1:1,000 BacLight red bacterial fluorescent stain (Invitrogen, B35001) in refractive index matching solutions (RIMS) (64). After a 1 h incubation at room temperature, the cells were imaged using a Leica Stellaris 5 confocal microscope on a 63×/1.4NA objective. The average fluorescent intensity of each sample was calculated using Imaris 10.2.0 image analysis software. A surface was created with the BacLight channel, and the average Cy3 fluorescence intensity per cell volume was calculated. Background intensity, as determined by a non-AHA treated control of each strain, was subtracted from the corresponding AHA-treated condition.

### DTT, antibiotic, and acidic condition induced aggregation assays

Cells were grown in TYEM until stationary phase before being treated with DTT and clarithromycin concentrations as indicated. For DTT assays (Fig. 5B), *M. smegmatis* mc^2^155 and Δ*whiB7* strains were grown aerobically in TYEM until after dispersal (48 h) and were either harvested immediately via the aggregation assay to produce a baseline for planktonic growth (T0) or treated with 2 mM DTT or an H2O control and incubated for 30 h before harvesting (78 h).

For clarithromycin assays (Fig. 6A), WT *M. smegmatis* was grown in TYEM until entrance into stationary phase (56 h). Cultures were harvested immediately (T0) or treated with 1 µg/mL or 2 µg/mL clarithromycin (+Clar1, +Clar2), or DMSO control, and incubated for 112 h before harvest (168 h). In parallel, some cultures received cells that were heat killed for 15 min at 98°C. These cells were pelleted and resuspended in 1 mL of spent media. Then, cultures received either 100 µL of resuspended cells (the equivalent of 1/10th of a heat killed culture), or 500 µL of cells pelleted again and resuspended in 100 µL spent media (the equivalent of ½ of a heat killed culture), or a 100 µL spent media control. These cultures were also incubated for 112 h before harvesting (168 h). Two MABS clinical isolates, 0253a and 0711a, were grown in TYEM until after dispersal (Fig. 6D and E) based on their growth kinetics (91 h or 119 h, respectively). Cultures were harvested immediately (T0) or received treatment: 80 mg/mL clarithromycin (+Clar80), 1 mM DTT (+DTT), or a combination of 1 mM DTT and 8 mg/mL Clar (+DTT +Clar8) or 1 mM DTT and 80 mg/mL Clar (+DTT +Clar80) and incubated before harvest at re-aggregation (168 h and 242 h, respectively).

For the effect of acidic conditions (Fig. S4), *M. smegmatis* was grown in TYEM until stationary phase (70 h). Cultures were treated with 1 M sterile HCl to a pH ~5.0 or 10 mM BME (reducing agent aggregation control). Tubes were harvested immediately (T0) or incubated for 67 h before harvesting.

### Minimum inhibitory concentration testing

WT *M. smegmatis* and *M. abscessus* clinical isolates 0253a and 0711a were tested for susceptibility to clarithromycin by the broth microdilution method (96). Briefly, 100 µL of TYEM was added to the first 11 columns of a 96 well flat bottom plate (Fisherbrand, Cat # FB012931), and 200 µL of 256 µg/mL clarithromycin was pipetted into the final column. Serial dilutions were performed, with 100 µL being transferred between wells with pipetting to mix, until the final 100 µL was ejected. TYEM cultures of WT *M. smegmatis* and *M. abscessus* clinical isolates 0253a and 0711a were diluted to an inoculum of 5 × 10^5^ CFU/mL (0.0005 OD_600_), and 100 µL of the inoculum was added to each well of the plate in triplicate per strain. The addition of inoculum brought the final clarithromycin concentration to a range of 0.0625 µg/mL to 128 µg/mL. MICs were read visually at Day 3 and Day 14 as recommended by the CLSI for testing rapid growing mycobacteria clarithromycin susceptibility.

### Statistical analysis

Statistical analysis was performed using Prism 10 (GraphPad, USA), and differences between treatment options were analyzed using Welch’s *t*-test for comparison of each group against T0. (*) indicates statistical significance with a *P*-value < 0.05; (**) indicates statistical significance with a *P*-value < 0.01, (***) indicates statistical significance with a *P*-value < 0.001, (****) indicates statistical significance with a *P*-value < 0.0001.

## AFTER EPUB

*[This article was published on 22 June 2026 with errors in the text. The errors were corrected in the current version, posted on 14 July 2026.]*

## SUPPLEMENTAL MATERIAL

Supplemental figures jb.00076-26-s0001.docx Figures S1 to S7.

Table S1 jb.00076-26-s0002.xlsx Strains, plasmids, and oligos used in this study.

Table S2 jb.00076-26-s0003.xlsx ΔΔCt analysis.

Table S3 jb.00076-26-s0004.xlsx MIC of clarithromycin on *M. smegmatis* and *M. abscessus*.

ASM does not own the copyrights to Supplemental Material that may be linked to, or accessed through, an article. The authors have granted ASM a non-exclusive, world-wide license to publish the Supplemental Material files. Please contact the corresponding author directly for reuse.
