# Supplementary material for: Activation of the antibiotic resistance factor WhiB7 can stimulate aggregate biofilm formation in stationary phase Mycobacterium smegmatis by reinitiating translation
Source: J Bacteriol. 2026 Jun 22;208(7):e00076-26. doi: 10.1128/jb.00076-26 (PMC13393426; doi:10.1128/jb.00076-26)
Supplement: Supplemental figures — Figures S1 to S7. [file jb.00076-26-s0001.docx]

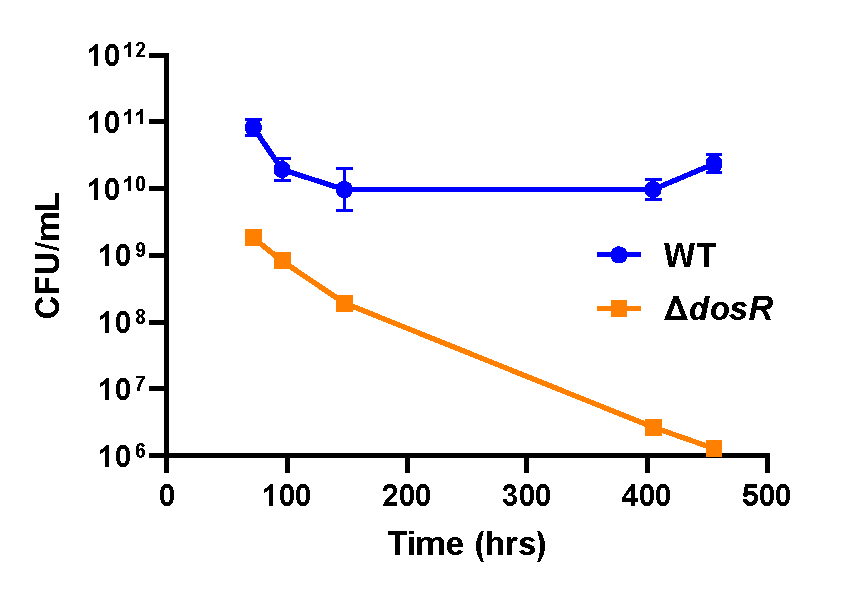


**Fig. S1 Unlike WT *M. smegmatis*, Δ*dosR* dies off under Wayne model hypoxia.**

WT *M. smegmatis* and a Δ*dosR* mutant grown in TYEM in Balch tubes under Wayne model hypoxia. CFUs were plated to monitor survival under hypoxia. CFUs were taken at 72, 96, 148, 405, and 456hrs past inoculation.


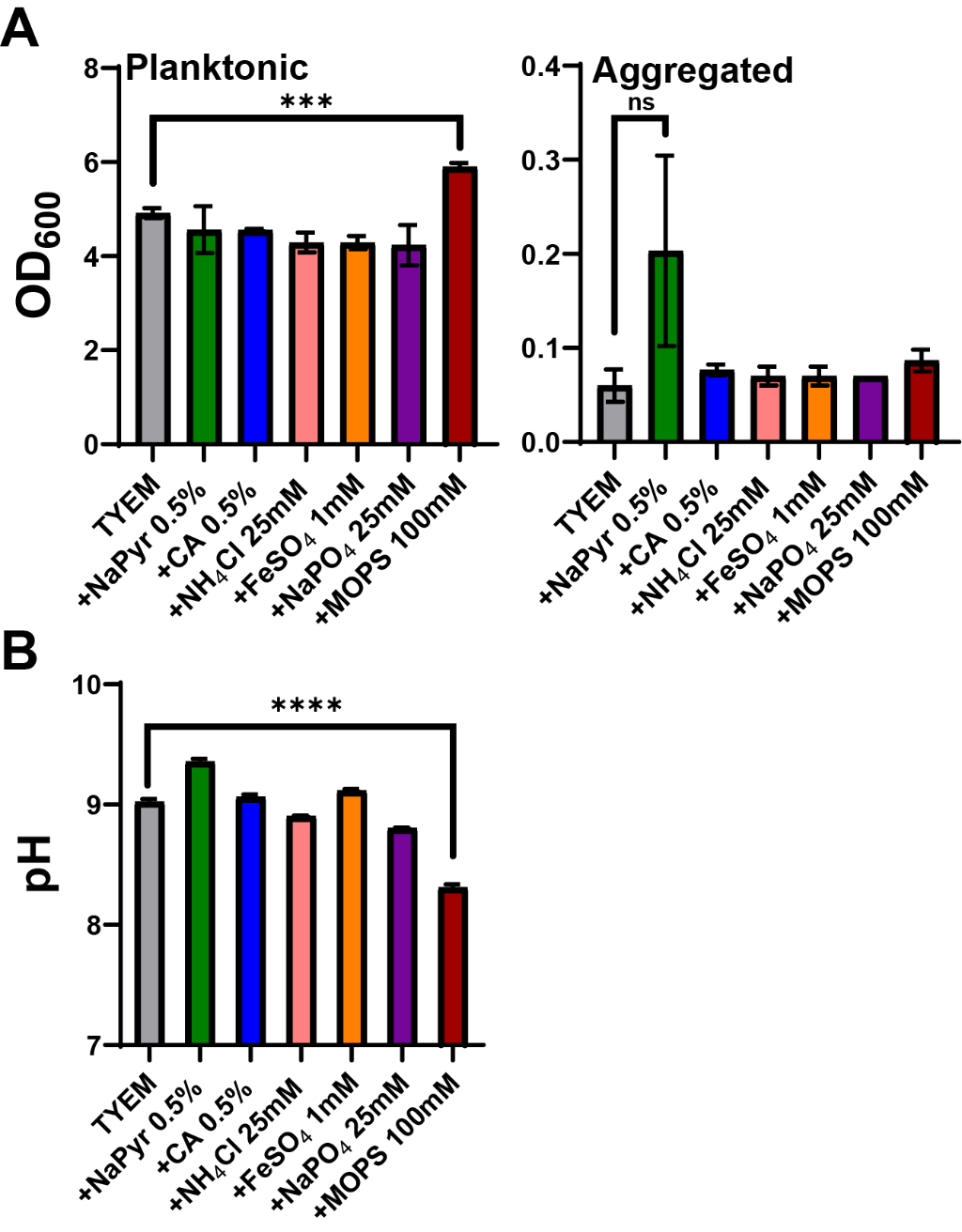


**Fig. S2 Culture alkylation and carbon limitation are major factors determining entrance into *M. smegmatis* stationary phase growth.**

(A) Aggregation assay in TYEM with the addition of nutrient supplements or buffered with MOPS. Cultures grew 108hrs before harvesting in triplicate. +NaPyr is the addition of pyruvate. +CA is addition of casamino acids. +NH_4_Cl is the addition of ammonium. +FeSO_4_ is the addition of iron. +NaPO_4_ was the addition of phosphate. +MOPS was the addition of MOPS buffer to neutralize changes to culture pH. Although statistically insignificant (p value > 0.13) addition of pyruvate increased aggregated growth while maintaining planktonic growth. The addition of MOPS prevented culture alkylation and increased planktonic growth. (B) pH of triplicate cultures of each condition at time of harvest.


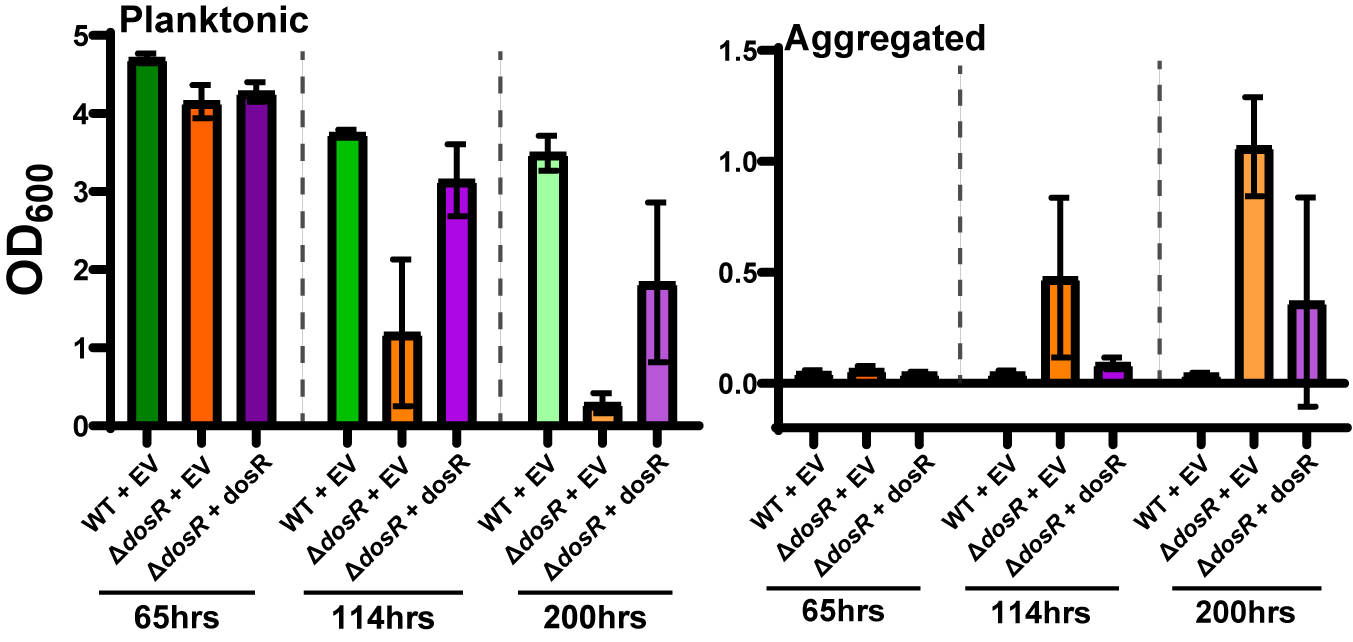


**Fig. S3 Complementation of *dosR* on the integrative plasmid pMH94 behind its native promoter partially restored WT *M. smegmatis* aggregation dynamics.**

Aggregation assay of WT *M. smegmatis* transformed with pMH94-EV and Δ*dosR* mutant transformed with pMH94-EV or pMH94-*dosR*. Cultures were grown in TYEM before harvesting in triplicate at indicated timepoints


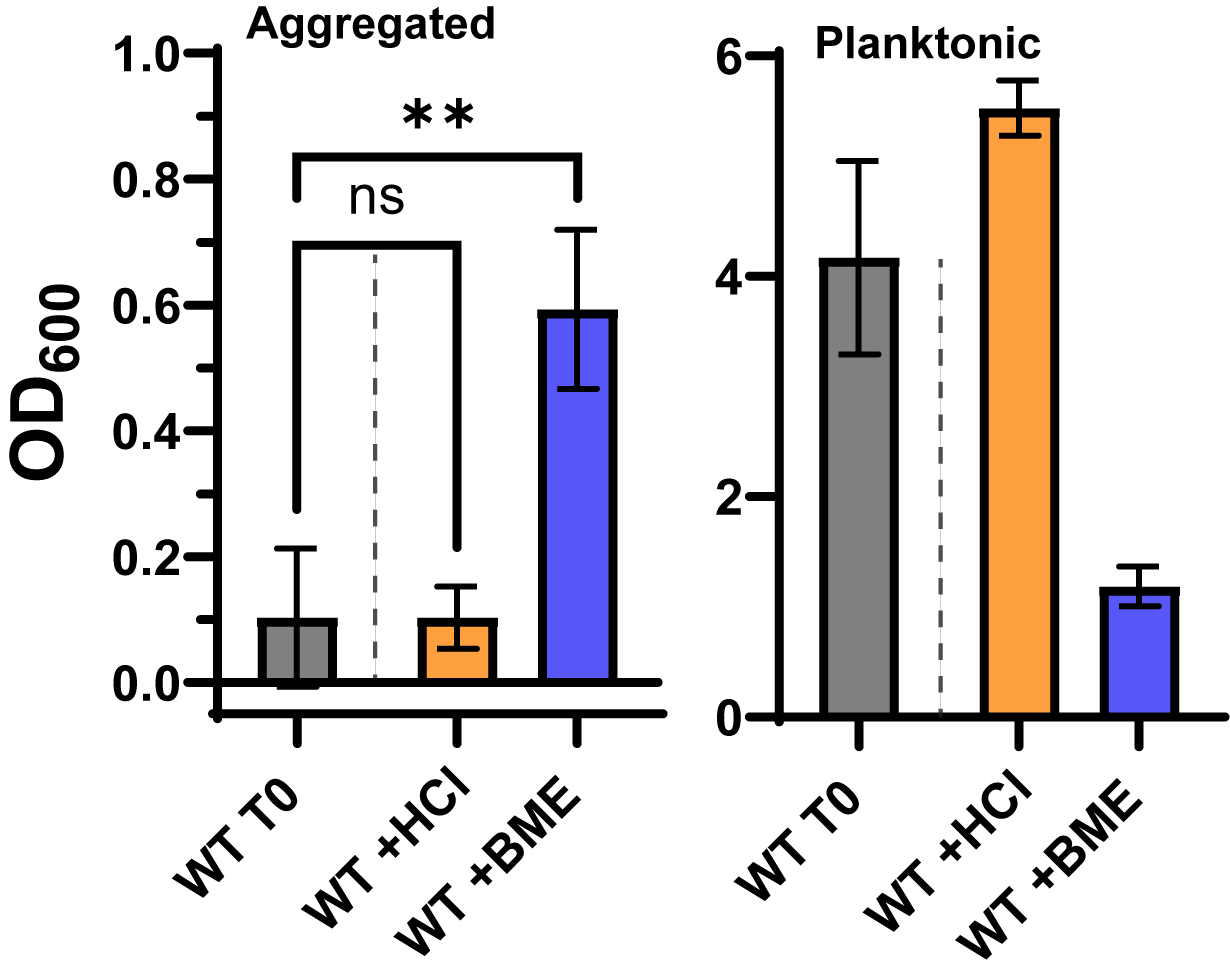


**Fig. S4** **Acid stress does not trigger re-aggregation in stationary phase *M. smegmatis.***

*M. smegmatis* grown in TYEM until treatment with 1M sterile HCl to a pH~5.0 or 10mM reducing agent BME after entrance to stationary phase (70hrs). Tubes were harvested immediately (T0) or incubated for 67hrs before harvesting.


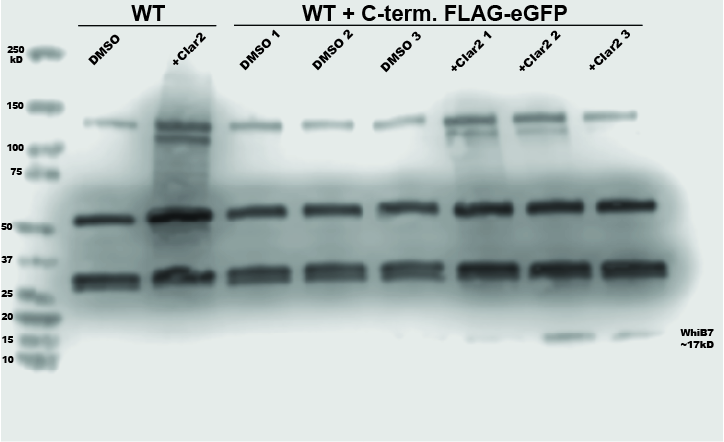


**Fig. S5 WhiB7 protein level increases after clarithromycin treatment.**

WT *M. smegmatis* with a *whiB7* C-terminal GFP-FLAG-6His-tag was grown in TYEM until 56hrs (post-dispersal). Cultures received 2µg/mL clarithromycin or a DMSO control. Cultures were harvested at 76hrs post treatment (132hrs post inoculation) and protein was extracted. Western blotting was performed using mouse anti-FLAG M2 antibody for the primary and HRP-coupled goat anti-mouse IgG antibody for the secondary. Clarithromycin treated FLAG-tagged samples showed an expected ~17kD band which was not present in the DMSO-treated control. Lane 1 untagged WT *M. smegmatis* treated with DMSO control. Lane 2 untagged WT *M. smegmatis* treated with 2µg/mL clarithromycin. Lanes 3,4,5 = three replicate samples of *whiB7* C-terminal-tagged *M. smegmatis* treated with DMSO control. Lane 6,7,8 = three replicate samples of *whiB7* C-terminal-tagged *M. smegmatis* treated with 2µg/mL clarithromycin.


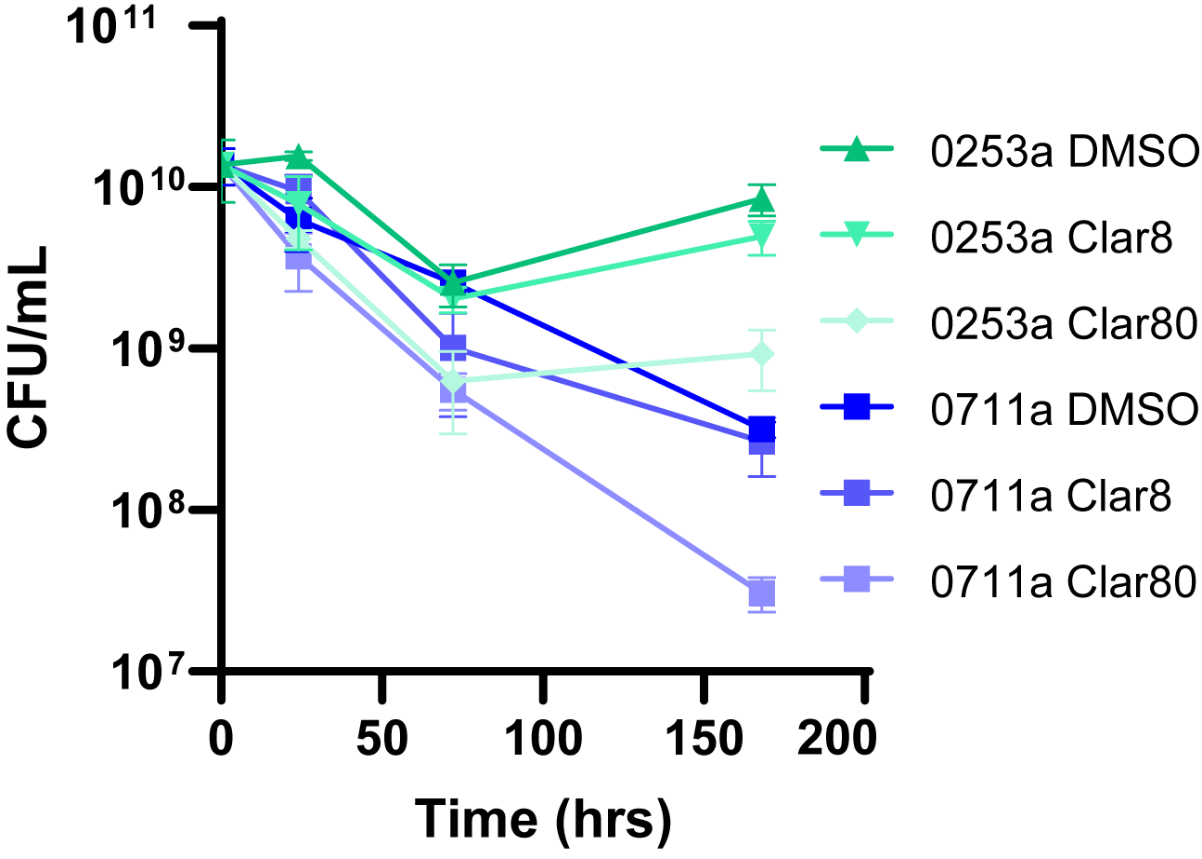


**Fig. S6** ***M. abscessus* CF clinical isolates 0253a and 0711a survival after treatment with clarithromycin.**

Cultures were grown in TYEM until after dispersal at 91hrs. CFUs were plated to establish baseline, and cultures received treatment with clarithromycin at 8 or 80µg/mL, or a DMSO control. CFUs were taken at 24hrs, 72hrs, 168hr post treatment (115hrs, 163hrs, 259hrs after inoculation).

**
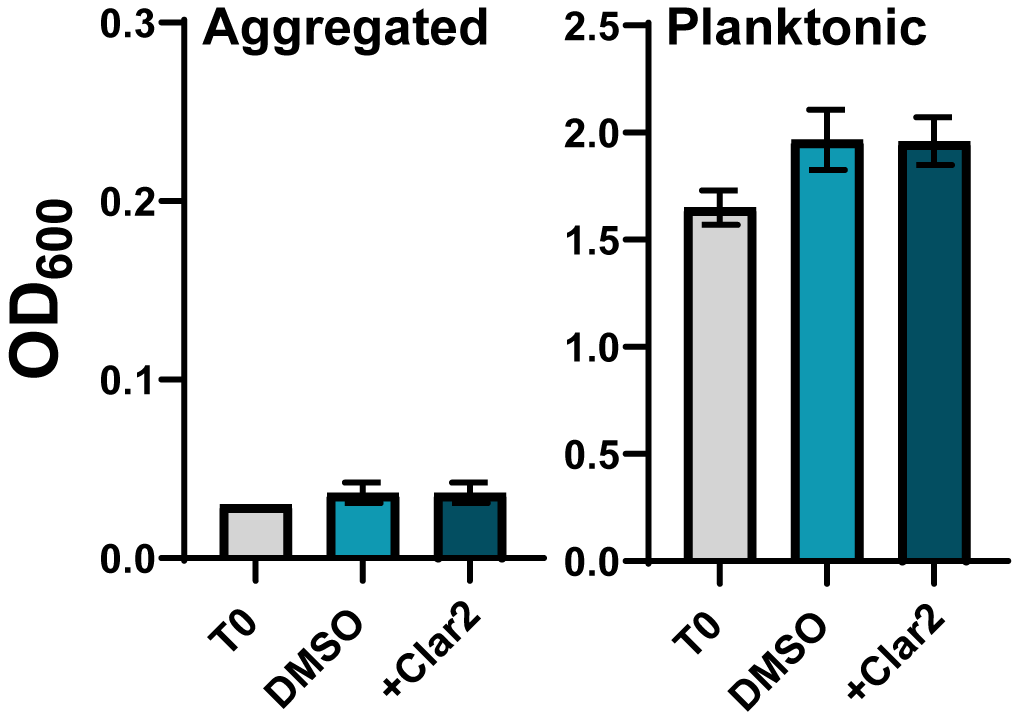
**

**Fig**. **S7** ***M. smegmatis* grown in the Wayne model does not re-aggregate after clarithromycin treatment.**

*M. smegmatis* grown in dilute TYEM under the Wayne model was treated with clarithromycin or a DMSO control. After dispersal at 56hrs, cultures were harvested immediately (T0) or treated with 2µg/mL clarithromycin to induce aggregation and incubated for 132hrs under anaerobic conditions before harvesting.
